# Nivolumab plus ipilimumab with or without live bacterial supplementation in metastatic renal cell carcinoma: a randomized phase 1 trial

**DOI:** 10.1038/s41591-022-01694-6

**Published:** 2022-02-28

**Authors:** Nazli Dizman, Luis Meza, Paulo Bergerot, Marice Alcantara, Tanya Dorff, Yung Lyou, Paul Frankel, Yujie Cui, Valerie Mira, Marian Llamas, Joann Hsu, Zeynep Zengin, Nicholas Salgia, Sabrina Salgia, Jasnoor Malhotra, Neal Chawla, Alex Chehrazi-Raffle, Ramya Muddasani, John Gillece, Lauren Reining, Jeff Trent, Motomichi Takahashi, Kentaro Oka, Seiya Higashi, Marcin Kortylewski, Sarah K. Highlander, Sumanta K. Pal

**Affiliations:** 1 Department of Medical Oncology, City of Hope Comprehensive Cancer Center, Duarte, CA USA; 2 Yale University School of Medicine, New Haven, CT USA; 3 Cettro Oncologia, Brasilia, Brazil; 4 Department of Immunology, Beckman Research Institute, City of Hope Comprehensive Cancer Center, Duarte, CA USA; 5 Division of Biostatistics, City of Hope Comprehensive Cancer Center, Duarte, CA USA; 6 The Translational Genomics Research Institute (TGen), Phoenix, AZ USA; 7 Miyarisan Pharmaceuticals, Co., Ltd., Tokyo, Japan

**Keywords:** Renal cell carcinoma, Cancer immunotherapy, Translational research

## Abstract

Previous studies have suggested that the gut microbiome influences the response to checkpoint inhibitors (CPIs) in patients with cancer. CBM588 is a bifidogenic live bacterial product that we postulated could augment CPI response through modulation of the gut microbiome. In this open-label, single-center study (NCT03829111), 30 treatment-naive patients with metastatic renal cell carcinoma with clear cell and/or sarcomatoid histology and intermediate- or poor-risk disease were randomized 2:1 to receive nivolumab and ipilimumab with or without daily oral CBM588, respectively. Stool metagenomic sequencing was performed at multiple timepoints. The primary endpoint to compare the relative abundance of *Bifidobacterium* spp. at baseline and at 12 weeks was not met, and no significant differences in *Bifidobacterium* spp. or Shannon index associated with the addition of CBM588 to nivolumab–ipilimumab were detected. Secondary endpoints included response rate, progression-free survival (PFS) and toxicity. PFS was significantly longer in patients receiving nivolumab–ipilimumab with CBM588 than without (12.7 months versus 2.5 months, hazard ratio 0.15, 95% confidence interval 0.05–0.47, *P* = 0.001). Although not statistically significant, the response rate was also higher in patients receiving CBM588 (58% versus 20%, *P* = 0.06). No significant difference in toxicity was observed between the study arms. The data suggest that CBM588 appears to enhance the clinical outcome in patients with metastatic renal cell carcinoma treated with nivolumab–ipilimumab. Larger studies are warranted to confirm this clinical observation and elucidate the mechanism of action and the effects on microbiome and immune compartments.

## Abstract (web summary)

A randomized trial in treatment-naive patients with metastatic renal cell carcinoma shows that the addition of a live bacterial product to an immunotherapy combination elicits promising clinical benefit in association with an enrichment of bacterial species, circulating cytokines and immune cell populations in responders.

## Main

Multiple groups have independently demonstrated a link between the gut microbiome and immunotherapy response in patients with cancer^1–4^. In patients with metastatic renal cell carcinoma (mRCC) and non-small cell lung cancer (NSCLC), Routy et al. evaluated the baseline stool microbiome profile prior to initiation of checkpoint inhibitors (CPIs) and identified multiple species (perhaps most notably *Akkermansia* spp.) that were associated with enhanced response rate and prolonged progression-free survival (PFS)^1^. Our group specifically assessed patients with mRCC and determined that species such as *Bifidobacterium adolescentis* and *Barnesiella intestinihominis* were associated with enhanced clinical benefit from CPIs^3^. Of note, other studies also support the role of *Bifidobacterium* spp. in the modulation of CPI response; in preclinical models, transplantation of fecal material enriched with *Bifidobacterium* spp. alone (even without CPIs) was sufficient to delay tumor growth^4,5^.

The dual CPI regimen of nivolumab (a programmed death-1 (PD-1) inhibitor) and ipilimumab (a cytotoxic T-lymphocyte-associated protein 4 (CTLA-4) inhibitor) represents a standard of care for the first-line treatment of mRCC^6,7^. Although multiple other options have emerged in recent years, combining vascular endothelial growth factor (VEGF)-directed therapy with PD-1 or programmed death-ligand 1 (PD-L1) inhibitors, the phase 3 dataset affirming the role of the nivolumab–ipilimumab combination as a first-line therapy has the longest follow-up to date^8–10^. In that study, patients were randomized either to that regimen or to sunitinib (a VEGF inhibitor); significant prolongation of PFS and overall survival was observed, with 42% of patients achieving a response (many of these durable)^11,12^. Those results imply, however, that the majority of patients receiving this regimen do not achieve a response; in fact, approximately 20% of patients have immediate progression of their disease on this regimen.

These results prompted prospective investigation of whether modulation of the gut microbiome could enhance the response to nivolumab–ipilimumab in patients with mRCC. The live bacterial product CBM588 contains *Clostridium butyricum*, a butyrate-producing anaerobic spore-forming bacterium^13–15^. In preclinical studies, the agent appears to be bifidogenic, possibly through expansion of interleukin (IL)-17A-producing γδ T cells and CD4 cells in the colonic lamina propria^13^. A retrospective study of patients with NSCLC receiving CPIs showed a profound impact of CBM588 on both PFS and overall survival^16^. The benefit of CBM588 appeared to be more pronounced in patients who had received antibiotic therapy, a striking finding given that antibiotics have consistently been shown to diminish the impact of CPIs^17^.

Based on these observations, we designed a randomized study to test prospectively the effects of CBM588 in patients with mRCC receiving nivolumab–ipilimumab. The primary endpoint of the study was the characterization of the effect of the agent on the relative abundance of gut microbial populations and specifically *Bifidobacterium* spp. Although it was not formally met, subgroup analyses showed an increase in *Bifidobacterium* spp. in patients who responded to CBM588 in combination with nivolumab–ipilimumab. Furthermore, despite low numbers, our data showed that patients receiving the live bacterial supplementation achieved higher objective response rates and prolonged PFS. Altogether, our findings support further evaluation of CBM588 in larger investigations.

## Results

### Patient characteristics

**Extended Data Fig. 1:**
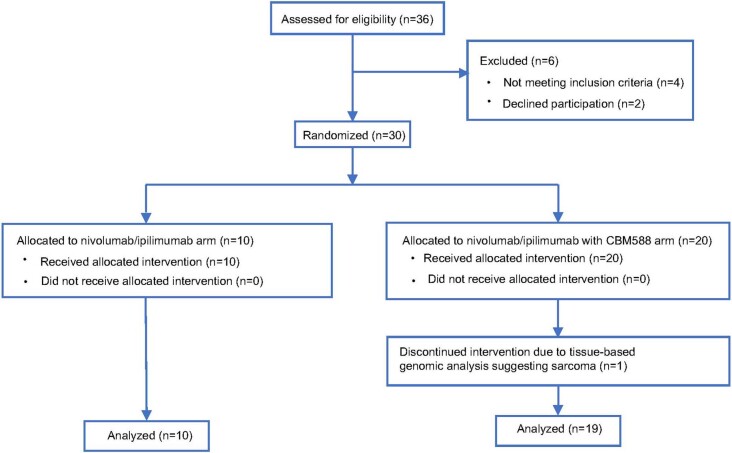
CONSORT diagram. Patient CONSORT diagram.

A total of 30 patients were randomized and started protocol-based treatment between 22 April 2019 and 30 December 2020 (Extended Data Fig. 1, CONSORT diagram). One patient originally randomized into the nivolumab–ipilimumab plus CBM588 arm was deemed ineligible after treatment initiation because tissue-based next-generation sequencing performed as part of routine clinical care showed genomic alterations pathognomonic for sarcoma. Ultimately, 29 patients were included in the final analysis. Baseline patient characteristics are listed in Table 1. The median age of the overall cohort was 66 years (range, 45–90 years) and the majority of the patients (72%) were male. Patients with sarcomatoid histology comprised 34% of the study cohort. The most common metastatic sites were lung, lymph nodes and bone. In the control arm, one patient cited consistent usage of a probiotic compound (yogurt fortified with *Bifidobacterium animalis*).

**Table 1:** Patient characteristics

	Nivolumab–ipilimumab (*n* = 10) Median (range) or *n* (%)	Nivolumab–ipilimumab plus CBM588 (*n* = 19) Median (range) or *n* (%)
Age (years)	64 (45–79)	66 (45–90)
Gender		
Male	8 (80)	13 (68)
Female	2 (20)	6 (32)
Race		
White	9 (90)	17 (89)
Asian	1 (10)	2 (11)
Ethnicity		
Non-Hispanic or non-Latinx	6 (60)	12 (63)
Hispanic or Latinx	4 (40)	7 (37)
Histologic subtype		
Clear cell	7 (70)	12 (63)
Clear cell with sarcomatoid features	2 (20)	5 (26)
Papillary with sarcomatoid features	1 (10)	1 (5)
Sarcomatoid dedifferentiation	–	1 (5)
IMDC prognostic risk		
Intermediate	7 (70)	17 (89)
Poor	3 (30)	2 (11)
Previous nephrectomy	4 (40)	9 (47)
Number of metastatic sites		
≥2	10 (100)	19 (100)
Most common metastatic sites		
Lung	6 (60)	13 (68)
Lymph node	7 (70)	8 (42)
Bone	4 (40)	7 (37)
Soft tissue	3 (30)	7 (37)
Liver	2 (20)	3 (16)
Pancreas	1 (10)	3 (16)

IMDC, International mRCC Database Consortium.

### Efficacy outcomes

**Fig. 1:**
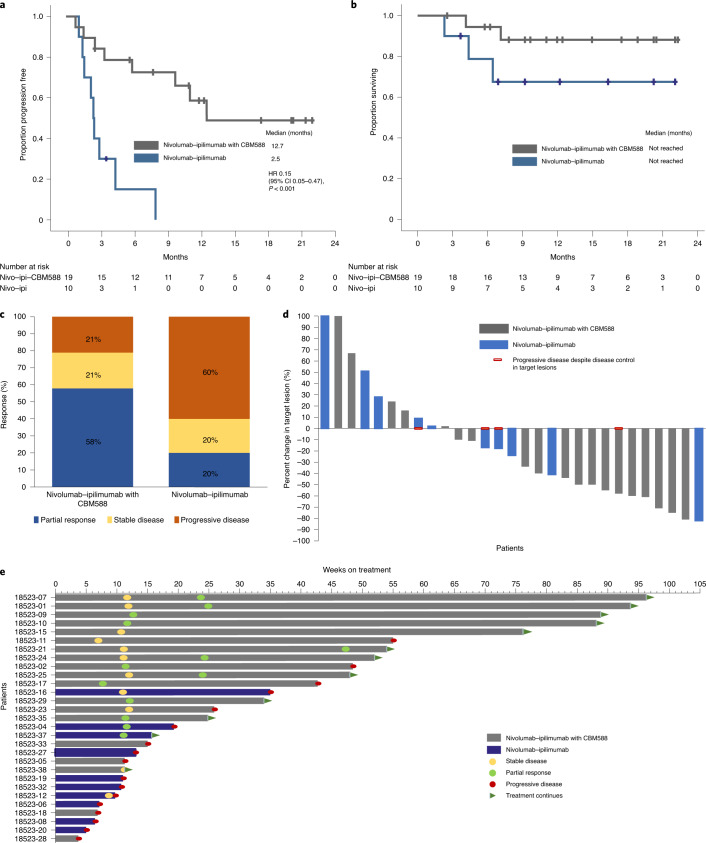
Efficacy outcomes in the treatment of patients with mRCC using nivolumab–ipilimumab with or without CBM588. **a**,**b**, Progression-free response (**a**) and overall survival (**b**). **c**–**e**, Best response by treatment arm (**c**), best change in target lesions from baseline (**d**), and a swimmers plot showing the response and survival characteristics (**e**). The data are from *n* = 29 patients (19 patients in the nivolumab–ipilimumab with CBM588 arm and 10 patients in the nivolumab–ipilimumab arm). The Kaplan–Meier log-rank test was used to compare survival between the two arms. Source data

The median follow-up at the time of data cut-off on 15 April 2021 was 12.2 months (95% confidence interval (CI) 10.6–13.8). At this time, 12 patients were still on treatment and 24 patients were alive. Median PFS was significantly prolonged in the nivolumab–ipilimumab plus CBM588 arm compared with the nivolumab–ipilimumab arm (12.7 versus 2.5 months, hazard ratio (HR) 0.15, 95% CI 0.05–0.47, *P* < 0.001; Fig. 1a). Median overall survival was not reached in both arms given that 83% of the study population was alive at the time of data cut-off (Fig. 1b).

A summary of best confirmed responses is presented in Fig. 1c. Objective responses were seen in 11 patients (58%) receiving nivolumab–ipilimumab plus CBM588 compared with two patients (20%) receiving nivolumab–ipilimumab (*P* = 0.06). A reduction in tumor target lesions was seen in 14 patients (74%) treated with nivolumab–ipilimumab plus CBM588 compared with five patients (50%) who received nivolumab–ipilimumab (Fig. 1d). As shown in Fig. 1e, the majority of responses were durable. At the time of data cut-off, no patients had a complete response. Disease control was achieved in 15 patients (79%) in the nivolumab–ipilimumab plus CBM588 arm and in four patients (40%) in the nivolumab–ipilimumab arm.

### Safety

Safety data are given in Table 2. Overall, 50% of the patients who received nivolumab–ipilimumab and 52% of the patients who received nivolumab–ipilimumab plus CBM588 had a grade 3 or 4 adverse event attributable to the treatment. Notable grade 3 or 4 toxicities observed in this series included fatigue, rash, adrenal insufficiency, hyperglycemia and diarrhea. Two patients required treatment discontinuation due to a treatment-related adverse event: one patient in the nivolumab–ipilimumab arm discontinued treatment due to grade 4 immune-related colitis and one patient in the nivolumab–ipilimumab plus CBM588 arm developed grade 3 immune-related transaminitis prompting treatment discontinuation. Both patients had complete recovery after corticosteroid therapy (the patient incurring immune-related transaminitis required additional therapy with mycophenolate). No treatment-related deaths occurred.

**Table 2:** Grade 2 or greater toxicities observed in ≥1 patient

	Nivolumab–ipilimumab (*n* = 10) *n* (%)			Nivolumab–ipilimumab plus CBM588 (*n* = 19) *n* (%)		
	Grade 2	Grade 3	Grade 4	Grade 2	Grade 3	Grade 4
Overall	2 (20)	5 (50)	0 (0)	6 (32)	9 (47)	1 (5)
Neutrophil count decreased	1 (10)					1 (5)
Fatigue		1 (10)		3 (19)	1 (5)	
Glucose intolerance		1 (10)		1 (5)	1 (5)	
Diarrhea		1 (10)		1 (5)	1 (5)	
Adrenal insufficiency				3 (19)	1 (5)	
Rash maculopapular				2 (11)	1 (5)	
Acute kidney injury		1 (10)		1 (5)	1 (5)	
Abdominal pain					1 (5)	
Alkaline phosphatase increase					1 (5)	
Acidosis					1 (5)	
Chest wall pain					1 (5)	
Pancreatitis					1 (5)	
Transaminitis		1 (10)		5 (26)	1 (5)	
Pruritus		1 (10)				
Dehydration		1 (10)				
Hypothyroidism	1 (10)			3 (19)		
Hyperthyroidism				3 (19)		
Arthralgia or myalgia				4 (22)		
Weight gain				2 (11)		

### Microbiome assessment

**Fig. 2:**
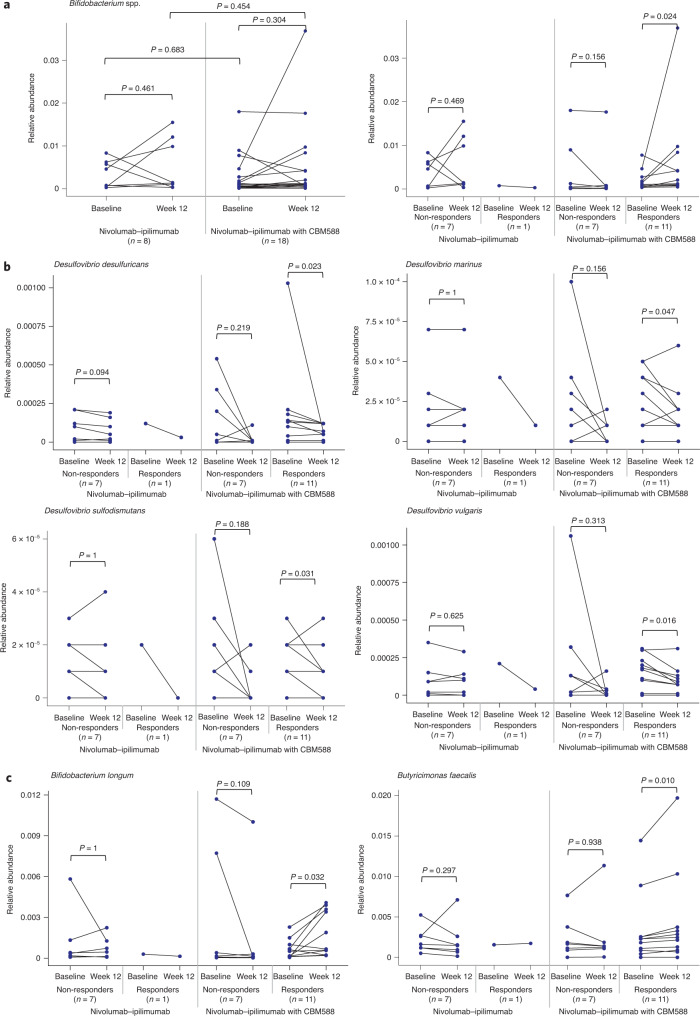
Microbiome assessment in patients with mRCC treated with nivolumab–ipilimumab with or without CBM588. **a**, Change in *Bifidobacterium* spp. from baseline to week 12 in patients by treatment arm, and by treatment arm and response. **b**,**c**, Decreases (**b**) and increases (**c**) in relative abundance of gut microbiome species associated with response to nivolumab–ipilimumab with CBM588. Analyses were performed using *n* = 52 stool samples from *n* = 26 patients (*n* = 18 patients in the nivolumab–ipilimumab with CBM588 arm and *n* = 8 patients in the nivolumab–ipilimumab arm). The Wilcoxon signed rank test was used to perform comparisons between two timepoints within the same treatment arm and the Mann–Whitney *U* test was used for comparisons between the two arms. Source data

**Extended Data Fig. 2:**
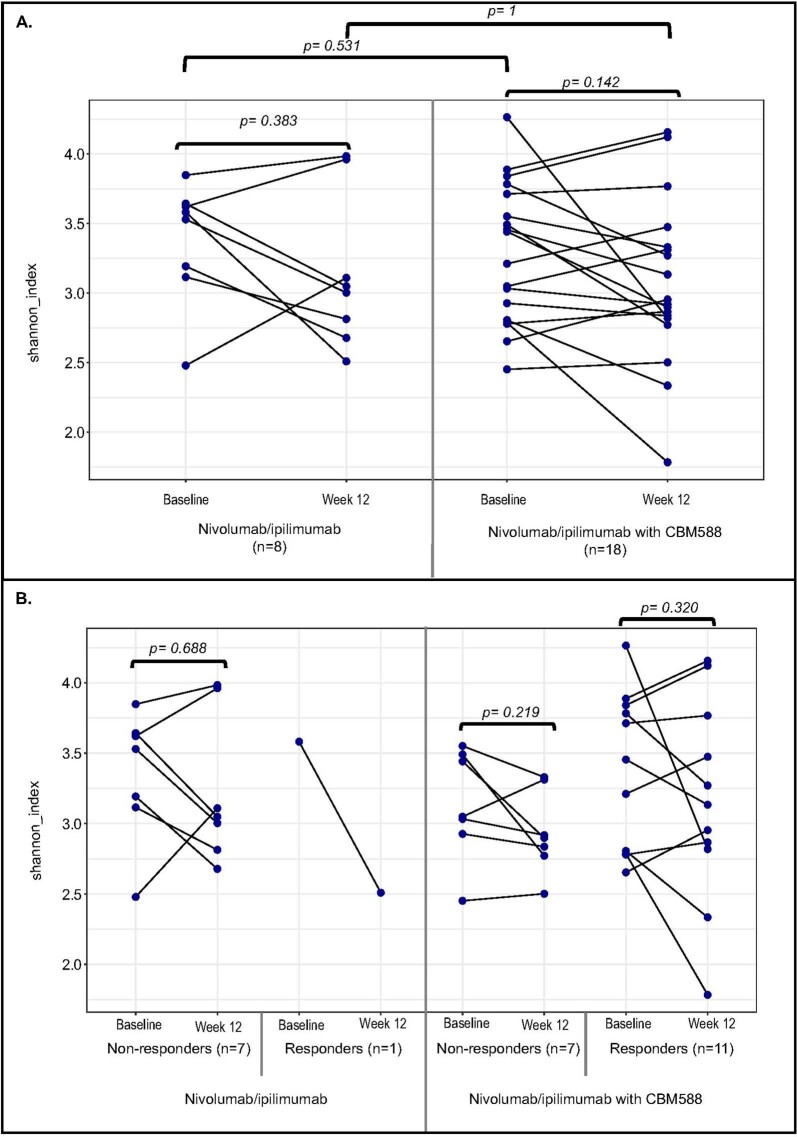
Assessment of Shannon diversity index by treatment arm and treatment arm and response. Assessment of Shannon diversity index by treatment arm (a) and treatment arm and response (b). Gut microbiome diversity was assessed using n=52 stool samples from n=26 patients (n=18 patients in nivolumab/ipilimumab with CBM588 arm [n=11 responders and n=7 non-responders]; and n=8 [n=7 non-responders and n=1 responder] patients in nivolumab/ipilimumab arm). Two-sided Mann-Whitney U test between nivolumab/ ipilimumab with CBM588 and nivolumab/ipilimumab. Two-sided Wilcoxon signed rank test between baseline and week 12. Shannon diversity index was calculated using the species-level abundance data (a,b). Source data

**Extended Data Fig. 3:**
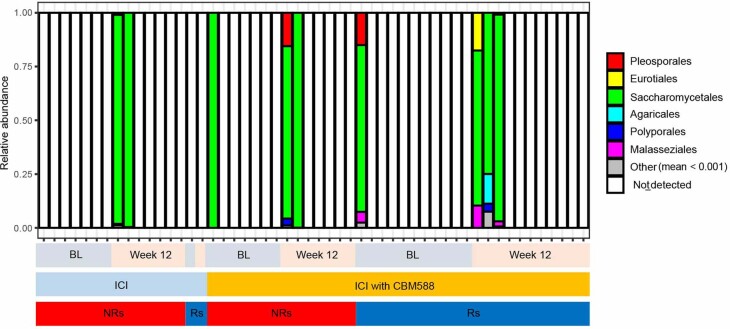
Assessment of fungal microbiome characteristics at order level. Fungal microbiome analyses were performed using n=52 stool samples from n=26 patients (n=18 patients in nivolumab/ipilimumab with CBM588 arm [n=11 responders and n=7 non- responders]; and n=8 [n=7 non-responders and n=1 responder] patients in nivolumab/ipilimumab arm). Taxa with a mean relative abundance less than 0.001 were summed as other. Source data

**Extended Data Fig. 4:**
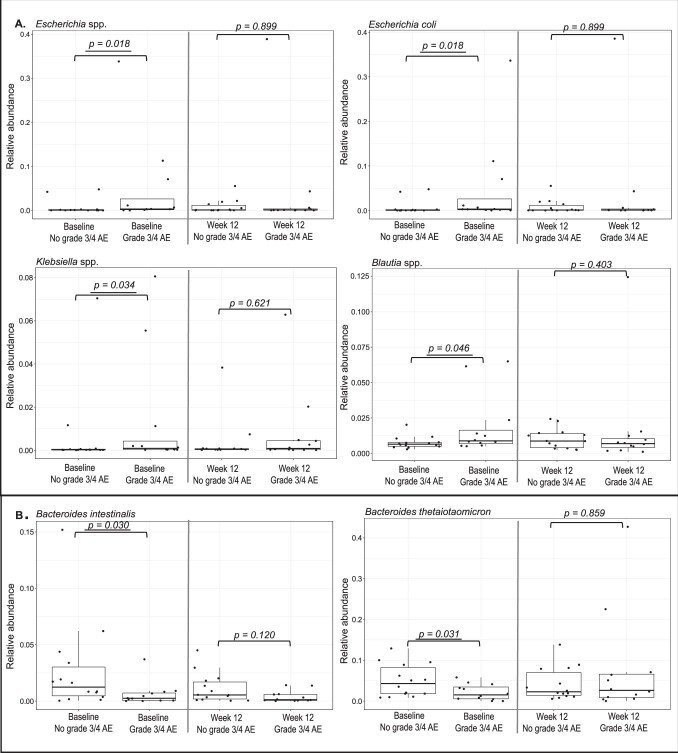
Differentially abundant taxa in patients who developed grade 3–4 adverse events (a) and those who did not develop grade 3–4 adverse events. Differentially abundant taxa in patients who developed grade 3–4 adverse events (a) and those who did not develop grade 3-4 adverse events (b). Analyses were performed using n=52 stool samples from n=18 patients in nivolumab/ipilimumab with CBM588 arm and n=8 patients in nivolumab/ipilimumab arm). Two-sided Mann-Whitney U test used to compare species across two groups. The length of the box plots represents the interquartile range (IQR) and the inside lines of the boxes represent the median. Source data

Baseline stool samples were collected from all patients enrolled in the study. Three patients (two in the control arm and one in the experimental arm) failed to submit a sample at week 12. A total of 52 samples from 26 patients were included in the gut microbiome analyses. There was no significant change in the relative abundance of *Bifidobacterium* spp. from baseline to week 12 (Fig. 2a), associated with nivolumab–ipilimumab with or without CBM588. These measures were not significant when using a natural log as a measure change or when using the Wilcoxon signed rank test. An exploratory subgroup analysis (using the Wilcoxon test) showed a statistically significant increase in *Bifidobacterium* spp. in patients receiving CBM588 and responding to treatment (*P* = 0.024; Fig. 2a). Further exploratory analyses also identified decreases in *Desulfovibrio* spp. in responders (Fig. 2b). In contrast, there was an increase in *Bifidobacterium longum* and *Butyricimonas faecalis* in the same group (Fig. 2c). There was no significant difference in Shannon diversity index between the baseline and week 12 samples in the nivolumab–ipilimumab arm or in the nivolumab–ipilimumab plus CBM588 arm, nor was there a significant difference in Shannon diversity index between the baseline samples in the two arms, or between the week 12 samples in the two arms (Extended Data Fig. 2). Fungal microbiome composition was assessed, but fungal species were detected in only 17% of the stool samples and no significant associations were observed between fungal microbiome characteristics and treatment response (Extended Data Fig. 3). In an exploratory analysis, an increased abundance of *Escherichia coli*, *Klebsiella* spp. and *Blautia* spp. was seen at baseline in patients who incurred grade 3 or 4 toxicities (Extended Data Fig. 4). These exploratory analyses were not adjusted for multiple comparisons and as such should be considered hypothesis generating.

**Fig. 3:**
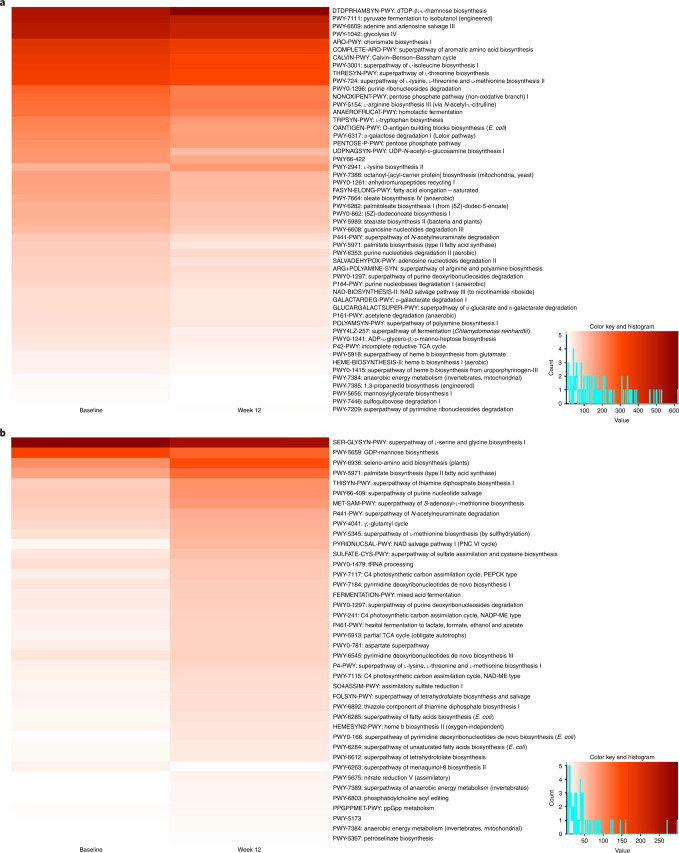
Changes in metabolic pathways in patients with mRCC treated with nivolumab–ipilimumab with or without CBM588. **a**,**b** Metabolic pathways with significantly different counts between baseline and week 12 in the nivolumab–ipilimumab with CBM588 arm (**a**) and the nivolumab–ipilimumab arm (**b**). Gut microbiome analyses were performed using *n* = 52 stool samples from *n* = 26 patients (*n* = 18 patients in the nivolumab–ipilimumab with CBM588 arm (*n* = 11 responders and *n* = 7 non-responders); and *n* = 8 patients (*n* = 7 non-responders and *n* = 1 responder) in the nivolumab–ipilimumab arm). The Wilcoxon signed rank test was used to compare metabolic pathways between the two timepoints. Source data

Analysis of metabolic pathways at baseline and week 12 yielded several notable findings. In detail, the dTDP-β-l-rhamnose biosynthesis, l-lysine biosynthesis II and superpathway of pyrimidine ribonucleosides degradation pathways were found to be upregulated after treatment with nivolumab–ipilimumab and CBM588 (*P* = 0.001, *P* = 0.007, *P* = 0.037, respectively). A total of 49 pathways were found to be downregulated in this patient cohort. In the nivolumab–ipilimumab arm, upregulation of 37 pathways and downregulation of three pathways were observed with treatment. Figure 3 shows heatmaps demonstrating the changes in metabolic pathways between baseline and week 12 by treatment arm.

### Assessment of circulating cytokines and immune cell populations

**Fig. 4:**
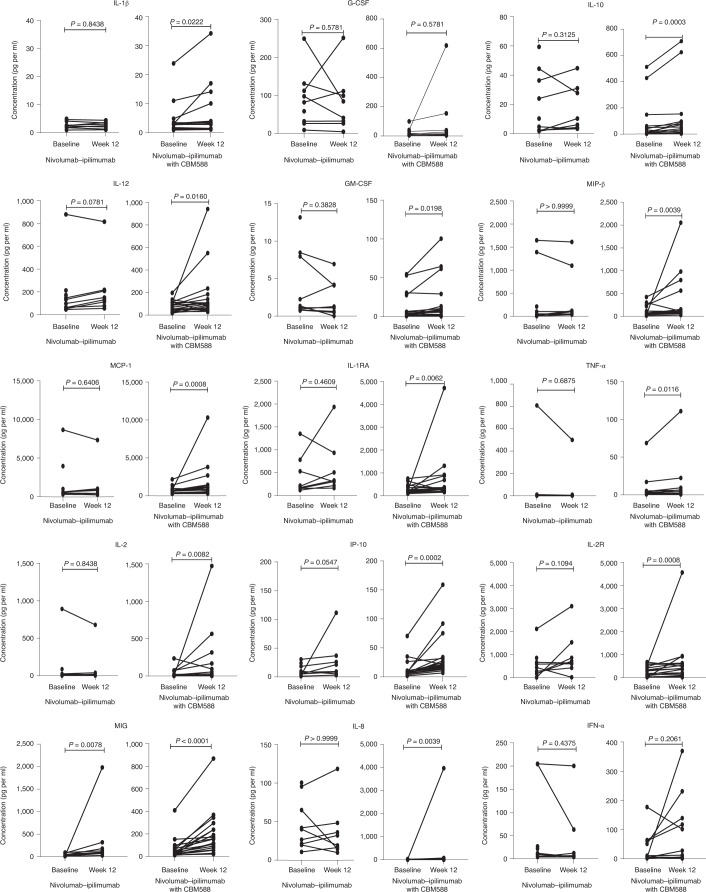
Changes in circulating cytokine levels from baseline to week 13 by treatment arm. Cytokine analyses were performed using *n* = 54 blood samples from *n* = 27 patients (*n* = 19 patients in the nivolumab–ipilimumab with CBM588 arm and *n* = 8 patients in the nivolumab–ipilimumab arm). Wilcoxon signed rank test was used to compare cytokine levels between the two timepoints. Source data

**Extended Data Fig. 5:**
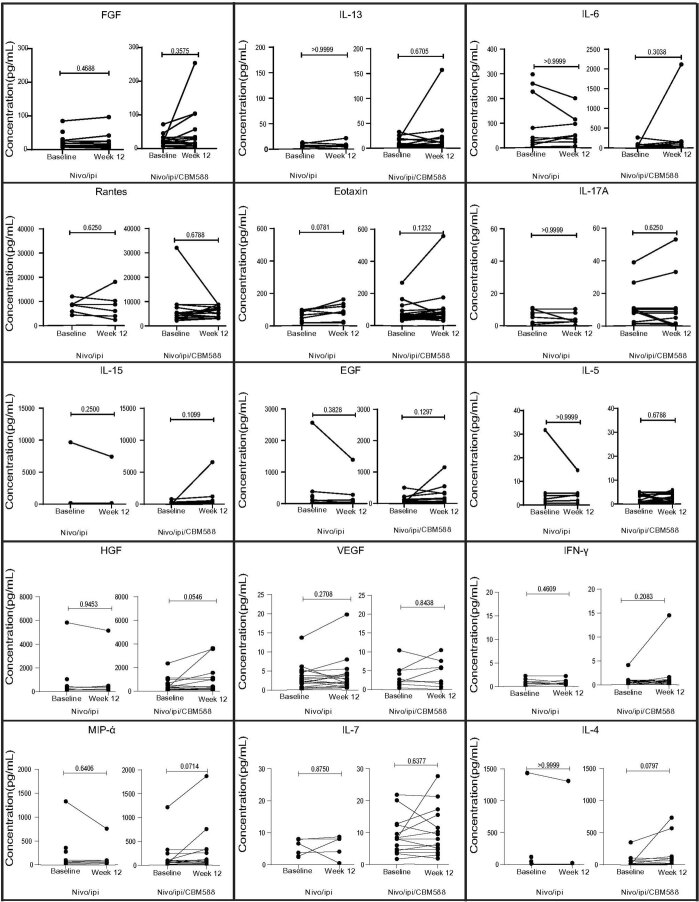
Changes in circulating cytokine levels from baseline to week 12 by treatment arm. Changes in circulating cytokine levels from baseline to week 12 by treatment arm. Cytokine analyses were performed using n=54 blood samples from n=27 patients (n=19 patients in nivolumab/ipilimumab with CBM588 arm and n=8 patients in nivolumab/ipilimumab arm). Wilcoxon signed rank test was used to compare cytokine levels across two timepoints. Source data

**Extended Data Fig. 6:**
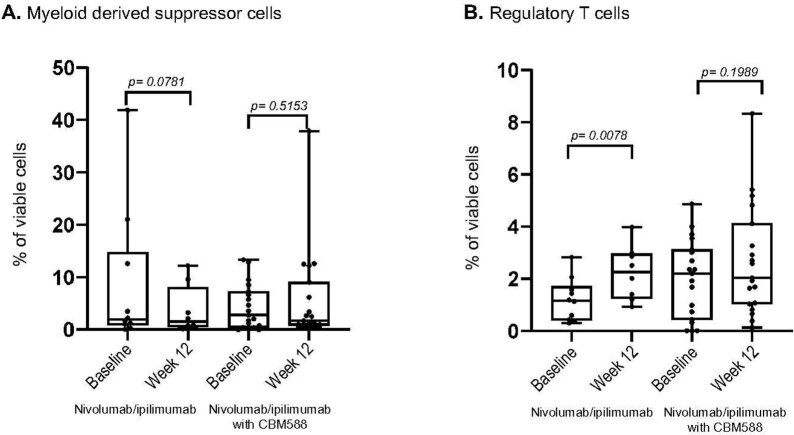
Changes in myeloid-derived suppressor cell and regulatory T cell populations by time in nivolumab/ipilimumab arm (a) and nivolumab/ipilimumab with CBM588 arm (b). Changes in myeloid-derived suppressor cell and regulatory T cell populations by time in nivolumab/ipilimumab arm (a) and nivolumab/ipilimumab with CBM588 arm (b). Immune cell populations were assessed in n=54 blood samples from n=27 patients (n=19 patients in nivolumab/ipilimumab with CBM588 arm and n=8 patients in nivolumab/ipilimumab arm). Wilcoxon test was used to compare immune cell populations at two timepoints. The length of the box plots represents the interquartile range (IQR) and the inside lines of the boxes represent the median. Whiskers (the vertical lines extending below and above each box) are used to represent the minimum and the maximum observation. Source data

Peripheral blood samples were collected at baseline and at weeks 7, 12, 17 and 25 of treatment. We elected to use baseline and week 12 (±4 weeks) samples for this analysis because these are the typical timepoints used in first response assessment. Two patients who discontinued treatment prior to week 12 sample collection were excluded. A total of 54 samples from 27 patients were available for the final analysis. Figure 4 and Extended Data Fig. 5 show the changes in circulating cytokine levels between baseline and week 12 by treatment arm. Of 31 cytokines evaluated, only the level of monokine induced by interferon-γ (MIG, also known as CXCL9) was found to increase in both the nivolumab–ipilimumab and the nivolumab–ipilimumab plus CBM588 arms with time (*P* = 0.0078 and *P* < 0.0001, respectively). Levels of IL-1β, granulocyte colony-stimulating factor (G-CSF), IL-10, IL-12, granulocyte–macrophage colony-stimulating factor (GM-CSF), macrophage inflammatory protein-β (MIP-β) (also known as CCL4), monocyte chemoattractant protein-1 (MCP-1), IL-1 receptor antagonist (IL-1RA), tumor necrosis factor-α (TNF-α), IL-2, interferon-γ-inducible protein 10 (IP-10), IL-2 receptor (IL-2R) and IL-8 were found to increase only in patients in the nivolumab–ipilimumab plus CBM588 arm (Fig. 4). No significant changes were observed in myeloid-derived suppressor cell between baseline and week 12 in both arms. Although there was a statistically significant increase in regulatory T cell populations from baseline to week 12 in patients treated with nivolumab–ipilimumab, such a change was not observed in patients treated with nivolumab–ipilimumab with CBM588 (Extended Data Fig. 6).

## Discussion

The results of this randomized clinical trial suggest that supplementation with live bacterial products may augment the activity of CPIs. Perhaps more importantly, the efficacy analyses highlighted a significant improvement in PFS with the addition of CBM588 to nivolumab–ipilimumab. Although this must be cautiously interpreted given the small sample size, consistent results were obtained favoring CBM588 in our analyses of response rate and overall survival. Also, although no significant change in *Bifidobacterium* spp. was observed with CBM588 therapy, we did observe an increase in these organisms specifically in responders.

The present findings reinforce several recent observations documenting synergy between immunotherapy and microbiome modulation. Baruch et al. recently reported a series of 10 patients with metastatic melanoma who were refractory to treatment with PD-1 inhibitors^18^. Treatment with fecal microbiota transplantation (FMT) (with feces derived from responding patients) yielded a response rate of 30%. FMT has a well-defined role in *Clostridium difficile* colitis, and an emerging role in inflammatory bowel diseases^19–21^. However, concerns have emerged over potential toxicities associated with this approach, with two patients having documented extended-spectrum beta-lactamase (ESBL)-producing *E.* *coli* bacteremia following transplantation^22^. As such, interest has turned to identifying the specific strains of bacteria that may increase responsiveness to CPIs. In a single-arm study including patients with triple-negative breast cancer, Francisco-Anderson et al. assessed EDP1503 (a single strain of *Bifidobacterium animalis lactis*)^23^. In 12 evaluable subjects, two partial responses were observed, including one response in a patient with prior exposure to checkpoint inhibition. The response rate of 18% seen in their study, albeit with a limited sample size, exceeds the response rate of 5% anticipated based on previously published data^24^.

The present data bolster a large, retrospective experience showing increased activity of CPIs in patients with NSCLC receiving CBM588^16^. Although not powered specifically to assess these endpoints, our study showed a significant advantage in PFS and a trend for improved overall survival with the addition of CBM588 to nivolumab–ipilimumab in patients with mRCC. When comparing the present results with the previously published data from CheckMate-214, the phase 3 study comparing nivolumab–ipilimumab with sunitinib, several differences in outcome are apparent. For instance, the response rate with nivolumab–ipilimumab in CheckMate-214 was 46%, compared with 22% in the control arm of the present study. Although this could simply be a byproduct of the small sample size, it is important to note that we had a large proportion of patients with bone metastases (38%) and many patients with synchronous metastatic disease with intact primary tumors (55%), both of which are strong adverse prognostic factors^25^. Additionally, an intriguing possibility is that dietary restrictions on the control arm could have influenced these results. Patients in the control arm were not allowed to consume bacteria-fortified foods, such as yogurt, or take any other supplements that might influence microbiome composition. Such practices were not prohibited in CheckMate-214. Notably, the one patient in the control arm who consumed yogurt fortified with *Bifidobacterium animalis* had the deepest response to therapy, with an 82% reduction in tumor dimensions.

Our exploratory analysis yielded insights into the mechanisms underlying the effect of CBM588 on the function of the gut microbiome. Notably, an upregulation of the dTDP-β-l-rhamnose biosynthesis pathway was observed in the nivolumab–ipilimumab with CBM588 arm. Rhamnose is a non-digestible carbohydrate that has a propionogenic (as opposed to butyrogenic) effect^26^. Like butyrate, propionate is also a short chain fatty acid that has been shown to have an anticancer effect: previous in vivo studies assessing CBM588 have demonstrated upregulation of this metabolite^27,28^. Furthermore, a decrease in the glycolysis IV pathway and in the pyruvate fermentation to isobutanol pathway, which are related to pyruvate processing, was also observed in the nivolumab–ipilimumab with CBM588 arm. This is somewhat predictable, given an anticipated increase in butyrate-consuming species and a resultant decrease in the dependence on glycolysis. In addition, the O-antigen building blocks biosynthesis (*E*. *coli*) pathway was also downregulated, which may be related to the inhibitory effect of CBM588 on enterohemorrhagic *E*. *coli*^29^. We also explored the potential association between CBM588 and systemic immune response. In this analysis, we saw significant increases in chemokines including CCL2 (MCP-1), CCL4 (MIP-1β), CXCL9 (MIG) and CXCL10 (IP-10) in patients receiving CBM588, but not in the control arm. These findings are corroborated by a recent study in melanoma in which on-treatment biopsies had increases in several of these mediators of dendritic cell and T cell recruitment with nivolumab and/or ipilimumab therapy^30^. Both CXCL9 and CXCL10, known as important for cytotoxic T cell and T helper 1 cell recruitment, can reportedly be upregulated by gut microbiota in patients with colorectal cancer^31^. Although there is some question as to whether our findings in blood are representative of the tumor microenvironment, a study of mRCC including patients receiving nivolumab suggested a correlation between chemokines such as CXCL9 and CXCL10 in simultaneously collected serum and on-treatment biopsy samples^32^. Our immune cell phenotyping suggested no significant change in myeloid-derived suppressor cell populations from baseline to 12 weeks in patients in either study arm. An increase in regulatory T cell populations was seen in the control arm but no change was seen in patients receiving CBM588, which may further support the immunomodulatory properties of CBM588.

The clinical observations in this randomized study are somewhat in agreement with the a priori hypothesis, suggesting that CBM588 (a butyrate-producing bacterial strain) would increase the abundance of species of *Bifidobacterium* spp. and thereby enhance immunotherapy response, given that the bifidogenic properties of CBM588 were noted to a greater extent in responders. This hypothesis is based on previous preclinical work showing that oral *Bifidobacterium* spp. could improve the activity of CPIs in murine models^5^. Curiously, this augmentation of response may be limited to CPIs: our previously published data suggest that certain species of *Bifidobacterium* (for example, *B.* *adolescentis*) may be associated with a lack of response to VEGF inhibition^33^. We are therefore in the process of devising a follow-up study comparing cabozantinib (a dual VEGF and MET inhibitor) with nivolumab with or without CBM588. This study will help us to understand whether a synergy exists between CBM588 and the combination of VEGF-directed therapies and a CPI, given that the latter is emerging as a front-line standard. Our work does not suggest any link between *Akkermansia* spp. and response; this is of importance given that multiple studies (including our own) have linked an abundance of *Akkermansia* spp. to CPI response^1,3^.

Although many studies have focused on the association between CPI response and microbiome profile, one recent study linked toxicity with dual CTLA-4 and PD-1 inhibition to an increased abundance of *Bacteroides intestinalis* in the stool of melanoma patients^34^. Our study was well positioned to explore this phenomenon, given that approximately half of the patients in both the control and experimental arms developed moderate–severe toxicity. Indeed, this exploratory analysis suggested several bacterial species (including *E.* *coli* and *Klebsiella* spp.) that were associated with toxicity. Interestingly, *B.* *intestinalis* was more common in patients who did not develop grade 3 or 4 events. The difference in findings could be due to many factors, such as the disease setting (melanoma versus renal cell carcinoma) and the limited sample size. Also, although the present study sought to augment the stool microbiome to enhance response, another potential therapeutic direction is the modulation of the gut and reduction of the species that may be linked to increased toxicity.

The present study faces the challenge of other projects in the microbiome space: although we were diligent in monitoring the diet of patients and carefully sought probiotic restriction in the control arm, we cannot account for the impact of other variations in dietary intake upon the results. Of note, we did not limit enrollment of patients who may have received recent antibiotic therapy. Interestingly, we identified only three patients who had received antibiotic therapy ≤14 days before the study treatment (a common exclusion criterion in clinical trials)^9,10^. Although existing data might suggest a diminished response to immunotherapy, each of these patients achieved a partial response^35^. Additionally, although our hypothesis is that it was the the bifidogenic properties of *C.* *butyricum* that drove the improvements in clinical outcome, one cannot rule out the possibility that changes in other species are responsible. For example, we saw consistent declines in *Desulfovibrio* spp. in responders; this genus has been associated with the pathogenesis of fatty-liver-associated liver cancer^36^. Preclinical models also suggest that these species may lower the burden of colorectal cancer through biofilm production and sulfate reduction^37^. Other limitations of the study include the small sample size, the lack of a placebo control, the absence of baseline tumor genomic profiling and long-term follow-up data for endpoints such as overall survival, and no planned assessment of patient-reported outcomes. Within the confines of the limited sample size, the intent was to increase the biologic and clinical utility of the findings by incorporating a control arm without CBM588 supplementation, and the 2:1 randomization schema was incorporated to increase the experience with combination therapy.

Despite these limitations, this study suggests that a live bacterial product may augment the activity of CPIs. Given what appears to be an acceptable safety profile, it is important to validate these findings in larger series and across different tumor types.

## Methods

### Patient eligibility

The study (NCT03829111) was approved by the US Food and Drug Administration and by the City of Hope Institutional Review Board. The study protocol is presented in the Supplementary Appendix. This was a single-center open-label investigator-initiated trial involving patients with histologically confirmed clear cell renal cell carcinoma and/or sarcomatoid histology. Patients had to be aged 18 years or older and have histologically confirmed mRCC with no prior systemic therapy (prior adjuvant therapy was allowed unless with a CPI). Patients were required to have intermediate- or poor-risk disease based on International mRCC Database Consortium (IMDC) criteria. Measurable disease by Response Evaluation Criteria in Solid Tumors (RECIST) version 1.1 was required. Inclusion and exclusion criteria are presented in the study protocol (Supplementary Appendix). Patients were counseled extensively that use of other probiotics, yogurt or any bacteria-fortified foods was not allowed while on the protocol.

Patients were required to supply written informed consent prior to participating. All study procedures were undertaken in accordance with the Declaration of Helsinki.

### Study design and treatment

Patients were randomized in a 2:1 fashion using a permuted block design to receive either nivolumab–ipilimumab with CBM588 or nivolumab–ipilimumab alone. Randomization was performed by the study statistician and the clinical investigator was notified of the treatment allocation only after randomization was completed. No stratification factors were used. In the control arm, patients received nivolumab at 3 mg per kg i.v. every 3 weeks and ipilimumab at 1 mg per kg i.v. every 3 weeks for 12 weeks, followed by nivolumab monotherapy at 480 mg i.v. monthly. Patients on the experimental arm received an identical schedule of nivolumab–ipilimumab but additionally received CBM588 at a dose of 80 mg orally twice daily. CBM588 was supplied by Miyarisan Pharmaceuticals and OSEL as a sachet (each sachet contained 40 mg of CBM588 powder), and the patients were instructed to mix the contents in an 8 oz glass of water and consume the slurry. Each 40 mg sachet formulation consisted of approximately 2.0 × 10^8^ c.f.u. of the viable active ingredient, C. *butyricum*, along with pharmaceutical excipients such as corn starch, calcium carbonate and lactose. Quality control tests of CBM588 demonstrated compliance with the pharmaceutical good manufacturing practices and United States Pharmacopeia. In brief, total aerobic bacteria and fungi (molds or yeast) counts were below 100 c.f.u. g^−1^ and 20 c.f.u. g^−1^, respectively, and the formulation did not contain any bile-tolerant Gram-negative bacteria, *E*. *coli*, *Salmonella*, *Pseudomonas* *aeruginosa*, *Staphylococcus* *aureus* and or fungi such as *Candida* *albicans*. Patients were asked to maintain a daily dietary log and indicate if they had consumed bacteria-fortified foods (for example, yogurt) or probiotics.

Consistent with the US Food and Drug Administration label for nivolumab and ipilimumab, no dose reductions were permitted for these agents. Treatment was continued until the occurrence of disease progression, unacceptable adverse events or patient withdrawal.

### Endpoints and assessments

Patients were required to undergo computed tomography of the chest, abdomen and pelvis at baseline; technetium bone scan and central nervous system imaging were performed as clinically indicated. Patients were assessed with imaging at 12 week intervals thereafter, with follow-up until the termination of protocol-based therapy or death. Safety evaluations were conducted at 3 week intervals for 12 weeks, followed by monthly evaluation. Radiographic response was assessed using RECIST version 1.1.

Stool was collected from patients at baseline and 12 weeks. Patients were provided with a stool collection kit (OMNIgene Gut; DNA Genotek); samples were mailed to TGen North within 24 hours of collection. We used the whole metagenome sequence instead of 16S ribosomal RNA gene amplicon sequencing because it provides more specific identification of species and enables analysis of the metabolic pathways and genes associated with the metagenome. DNA was extracted from stool samples using the MagMax PowerMicrobiome extraction kit and the KingFisher Flex magnetic purification system (ThermoFisher) with prior bead beating using a TissueLyser (Qiagen). Bacterial load and fungal load were quantified using the BactQuant TaqMan assay and FungiQuant TaqMan assay, respectively^38,39^. Whole metagenome libraries were constructed using a KAPA HyperPrep Library Kit (Roche), and normalized, pooled and sequenced on the Illumina NextSeq platform (2 × 150 bp). Reads were trimmed using Trimmomatic to remove adapters and low-quality bases and reads^40^. Samples that passed quality assurance were taxonomically profiled using Kraken 2 v2.1.1^41^ and Bracken v2.5^42^ 3.0 and output was merged to retain species-level assignments.

To identify the functional potential of microbial communities, we ran MetaPhlAn 3.0 data through HUMAnN 3.0^43^. Generated metabolic pathways were compared using the Wilcoxon signed rank test between baseline and week 12 in the nivolumab–ipilimumab and nivolumab–ipilimumab plus CBM588 arms, separately. Metabolic pathways with a *P* value less than 0.05 were considered significant and were shown as a heatmap using the function heatmap.2 of the gplots package for R version 4.1.1.

Peripheral blood samples were collected in 10 ml cell preparation tubes (BD Biosciences) at baseline and weeks 7, 12, 17 and 25. All samples were processed within a window of 4–6 h after collection. Processing involved centrifugation at 1,800 ×*g* for 20 min followed by plasma extraction for circulating cytokine analysis. After plasma extraction, the remaining cell suspension was transferred to conical propylene tubes, washed in cRPMI and recentrifuged at 250 ×*g* for 7 min at room temperature (20° C) for isolation of peripheral blood mononuclear cells (PBMCs). PBMCs were then immersed in a mixture of PBS, FCS and sodium azide with Fc III/IIR-specific antibody (commercially available Fc III/IIR-specific antibodies that have been validated by BD and Biolegend) to block non-specific binding and the cells stained with viability dye and different combinations of fluorochrome-coupled antibodies to CD3, CD4, CD8, intracellular FoxP3, CD33, HLA-DR and CD15 (BD Biosciences). Flow cytometry data were collected using BD Fortessa and Cytek Aurora (Becton Dickinson and Cytek) and analyzed using FlowJo (Becton Dickinson)^44^. The plasma cytokine panel used in this study included IL-1RA, IL-1b, IL-2, IL-2R, IL-4, IL-5, IL-6, IL-7, IL-8, IL-10, IL-12, IL-13, IL-15, IL-17, Eotaxin, EGF, FGF, G-CSF, GM-CSF, IFN-α, IFN-γ, CXCL9, CXCL10, CCL2, CCL3, CCL4, RANTES, TNF-α and VEGF. Cytokine concentration was measured using the Cytokine 30-plex Human Panel run on the Luminex FLEXMAP 3D System. Changes in circulating cytokine levels and in regulatory T cell and myeloid-derived suppressor cell populations over time were compared across arms to examine the effects of CBM588 on the immune system.

### Statistical analysis

The primary objective of the study was to determine the change in *Bifidobacterium* spp. collected from baseline to 12 weeks. With a cumulative sample size of 30 patients (randomized in a 2:1 fashion), we would have 80% power to detect a 1 s.d. change in specific *Bifidobacterium* spp. between the study arms using a *t*-test with a one-sided type I error of 0.05. Secondary measures included comparison of the Shannon diversity index at baseline and at 12 weeks and quantitative comparisons of changes in the abundance of other specific bacterial species. Details of this analysis can be found in the full protocol (Supplementary Appendix). For exploratory analysis comparing the gut microbiome composition, cytokines, regulatory T cell and myeloid-derived suppressor cell populations between two timepoints, we used the Wilcoxon signed rank test due to the non-normal distribution of the data and for analyses comparing these variables between study arms, we used Mann–Whitney *U* test due to non-normal distribution of the data. Cytokine and immune cell populations were analyzed using GraphPad Prism version 8.4.2.

With respect to clinical endpoints, PFS was characterized as the time from randomization to disease progression or death (whichever occurred first), and overall survival was defined as the time from randomization to death. These were compared between the study arms using the Kaplan–Meier method and log-rank test. Objective response rate (complete or partial response) was compared between arms using the Fisher exact test. Disease control rate was assessed, reflecting the proportion of patients with either complete or partial response or stable disease as a best response to therapy. Clinical data were analyzed using SPSS version 21.0.

### Reporting Summary

Further information on research design is available in the Nature Research Reporting Summary linked to this article.

## Online content

Any methods, additional references, Nature Research reporting summaries, source data, extended data, supplementary information, acknowledgements, peer review information; details of author contributions and competing interests; and statements of data and code availability are available at 10.1038/s41591-022-01694-6.

## Supplementary information

Supplementary Appendix Final study protocol and data transfer agreement.

Reporting Summary

## Data Availability

Metagenomic data from stool sufficient to replicate the analyses presented herein will be deposited at the Translational Genomics Research Institute (TGen) and will be available upon request. The authors defer depositing the participant genomic data in national and international public repositories due to institutional policies and the absence of statements in patient consent forms that would have allowed controlled access distribution and genomic data availability. De-identified individual participant whole metagenome libraries and clinical data that underlie the results reported in this article are available for transfer on a specific secure server housed at TGen. Interested investigators can obtain and certify the data transfer agreement (DTA) and submit requests to the principal investigator, S.K.H. Proposals will be vetted by the TGen Data Access Committee. Investigators and institutions who consent to the terms of the DTA form, including but not limited to the use of these data for the purpose of a specific project and only for research purposes, and to protect the confidentiality of the data and limit the possibility of identification of participants in any way whatsoever for the duration of the agreement, will be granted access. TGen will then facilitate the transfer of the requested de-identified data. This mechanism is expected to be via an Aspera High Speed File Transfer Server but TGen reserves the right to change the specific transfer method at any time, provided appropriate levels of access authorization and control can be maintained. Source data are provided with this paper.

## Source data

Source Data Fig. 1 Clinical outcomes figure.
Source Data Fig. 2 Statistical source data.
Source Data Fig. 3 Statistical source data.
Source Data Fig. 4 Statistical source data.
Source Data Extended Data Fig. 2 Statistical source data.
Source Data Extended Data Fig. 3 Statistical source data.
Source Data Extended Data Fig. 4 Statistical source data.
Source Data Extended Data Fig. 5 Statistical source data.
Source Data Extended Data Fig. 6 Statistical source data.

## Extended data

are available for this paper at 10.1038/s41591-022-01694-6.
