# Supplementary material for: Nivolumab plus ipilimumab with or without live bacterial supplementation in metastatic renal cell carcinoma: a randomized phase 1 trial
Source: Nat Med. 2022 Feb 28;28(4):704–12. doi: 10.1038/s41591-022-01694-6 (PMC9018425; doi:10.1038/s41591-022-01694-6)
Supplement: Supplementary file 1 — Final study protocol and data transfer agreement. [file 41591_2022_1694_MOESM1_ESM.pdf]

nature portfolio

https://doi.org/10.1038/s41591-022-01694-6

Supplementary information

Nivolumab plus ipilimumab with or without live bacterial supplementation in metastatic renal cell carcinoma: a randomized phase 1 trial

In the format provided by the authors and unedited

SUPPLEMENTAL APPENDIX

This section contains the final protocol and the Data Transfer Agreement

CITY OF HOPE NATIONAL MEDICAL CENTER
1500 E. DUARTE ROAD
DUARTE, CA 91010

DEPARTMENT OF MEDICAL ONCOLOGY AND THERAPEUTICS RESEARCH

TITLE: **Pilot study to evaluate the biologic effect of CBM588 in combination with nivolumab/ipilimumab for patients with metastatic renal cell carcinoma**

CITY OF HOPE PROTOCOL NUMBER/VERSION: IRB # 18523 **VERSION: 05**

DATE(S)/ OF AMENDMENT(S)/REVISION(S):

COH Initial Review	Protocol Dated 12/28/2018	Version 00
Amendment 00	Protocol Dated 01/31/2019	Version 00
Amendment 01	Protocol Dated 07/02/2019	Version 01
Amendment 02	Protocol Dated 09/20/2019	Version 02
Amendment 03	Title Page Dated 10/30/2019	Version 03
Amendment 04	Protocol Dated 5/21/2020	Version 04
Amendment 05	Protocol Dated 6/12/2020	Version 05

**SPONSOR/IND NUMBER**	City of Hope/Osel, Inc/IND 18765
**SITE:**	Renal Cell Carcinoma
**STAGE (If applicable):**	IV
**MODALITY:**	Pilot, Immunotherapy
**TYPE:**	Therapeutic
**PRINCIPAL INVESTIGATOR:**	Sumanta Pal, MD
**COLLABORATING INVESTIGATOR(S):**	Paul Frankel, PhD<br>Sierra (Min) Li, PhD<br>Sarah Highlander, PhD (TGen)<br>Marcin Kortylewski, PhD
**PARTICIPATING CLINICIANS:**	Tanya Dorff, MD
**Research Coordinator**	JoAnn Hsu<br>Yung Lyou, M.D.

EXPERIMENTAL DESIGN TABLE

The study will employ randomization (2:1) to nivolumab/ipilimumab with CBM588 or nivolumab/ipilimumab alone.

Treatment Arms

Dose Level	Number of Patients	Dose		
		CBM588 All Cycles	Nivolumab/Ipilimumab Cycles 1-4	Nivolumab Cycles 5 +
**Arm 1**	**10**	None	3 mg/kg / 1 mg/kg	480 mg
**Arm 2**	**20**	80 mg bid	3 mg/kg / 1 mg/kg	480 mg

PROTOCOL SYNOPSIS

Protocol Title

Pilot study to evaluate the biologic effect of CBM588 in combination with nivolumab/ipilimumab for patients with mRCC

Study Description

The landscape of therapy for mRCC has changed drastically over the past decade. Most recently, nivolumab/ipilimumab has been introduced in the front-line setting based on data from the CheckMate214 trial, showing an overall survival benefit relative to suntinib. Although the data are encouraging for this strategy of dual checkpoint inhibition, the vast majority of patients are not cured of their disease. Only a minority (42%) of patients respond to immunotherapy, and approximately 20% of patients will progress through therapy with no clinical benefit. Recent studies suggest that the gut microbiome may play a key role in modulating responses to immunotherapy. Our preliminary data from patients receiving immunotherapy for mRCC have shown that certain gut bacteria (e.g., *Bifidobacterium*) may predispose to response. We therefore propose assessing CBM588, a live biotherapeutic, in combination with nivolumab/ipilimumab. CBM588 is a strain of *Clostridium butyricum*, and it has been shown that butyric acid bacteria have immunomodulatory and anti-inflammatory effects on the intestinal epithelium, and can restore species such as *Bifidobacterium spp* and *Lactobacillus spp* to the gut. This initial study of the combination will identify the biologic effect of CBM588 with nivolumab/ipilimumab, with the intent of driving further studies to assess augmentation of response.

Objectives

<u>Primary Objectives</u>

- (1) To determine the effect of CBM588 (in combination with nivolumab/ipilimumab) in modulation of the gut microbiome in patients with mRCC

<u>Secondary Objectives</u>

- (1) To evaluate the effect of CBM588 on the clinical efficacy of the nivolumab/ipilimumab combination
- (2) To determine the effect of CBM588 on systemic immunodulation of the nivolumab/ipilimumab combination in patients with mRCC
- (3) To determine the effect of CBM588 on toxicities such as diarrhea and nausea using CTCAE v5 criteria with the nivolumab/ipilimumab combination in patients with mRCC

Evaluation Criteria and Endpoints

<u>Primary Endpoint</u>

- (1) Change in *Bifidobacterium* composition of stool from baseline to week 12 of therapy on the CBM588+nivolumab/ipilimumab vs nivolumab/ipilimumab alone.

<u>Secondary Endpoints</u>

- (1a) Comparison of the Shannon index (a measure of microbial diversity) from baseline to week 12 of therapy on the CBM588+nivolumab/ipilimumab vs nivolumab/ipilimumab alone.
- (1b) Best overall response, by RECIST criteria, with nivolumab/ipilimumab alone vs nivolumab/ipilimumab with CBM588
- (1c) Progression-free survival (PFS), assessed as the duration of time from enrollment to progression, with nivolumab/ipilimumab alone vs nivolumab/ipilimumab with CBM588

- (2a) Comparison of the proportion of circulating Tregs at baseline to levels of circulating Tregs with nivolumab/ipilimumab alone vs nivolumab/ipilimumab with CBM588
- (2b) Comparison of the proportion of circulating MDSCs with nivolumab/ipilimumab alone versus nivolumab/ipilimumab with CBM588
- (2c) Comparison of IL-6, IL-8 and other cytokines/chemokines with nivolumab/ipilimumab alone versus nivolumab/ipilimumab with CBM588
- (3) Comparison of toxicities such as diarrhea and nausea using CTCAE v5 criteria with nivolumab/ipilimumab alone versus nivolumab/ipilimumab with CBM588

**Study Details**	
**Phase:**	Phase I
**Study Population:**	• Age 18 or greater<br>• Histologically confirmed mRCC with clear cell histology<br>• Advanced, metastatic disease<br>• Planned treatment with nivolumab/ipilimumab<br>• No prior therapy with immune checkpoint inhibition
**Sample Size**	30 patients
**Accrual Duration:**	2 years
**Participant Duration:**	12 months
**Study Duration**	3 years
**Sites/Facilities Enrolling Participants:**	City of Hope
**Study Agents:**	CBM588
**Sponsor:**	**Error! No text of specified style in document.**
**Industry Partner:**	Osel, Inc.
**Industry Partner Protocol ID:**	N/A

TABLE OF CONTENTS

SECTION		PAGE
Protocol Team		1
Experimental Design Schema		3
Protocol Synopsis		4
Table of Contents		6
List of Tables and Figures		7
Abbreviations		7
1.0	Objectives and Endpoints	8
1.1	Primary Objectives	8
1.2	Exploratory Objective	8
2.0	Background	9
2.1	Nivolumab/Ipilimumab for mRCC	8
2.2	The Microbiome in mRCC	9
2.3	CBM588	10
2.4	Overview and Rationale of Study Design	9
3.0	Eligibility Criteria	12
3.1	Inclusion Criteria	13
3.2	Exclusion Criteria	13
4.0	Participant Recruitment, Enrollment, and Randomization	15
5.0	Treatment Program	15
5.1	Treatment Program Overview	15
5.2	Cycle Definition	16
5.3	Treatment Plan	16
5.4	Agent Administration	16
5.5	Assessments and Special Monitoring	16
5.6	Duration of Therapy and Criteria for Removal from Protocol Therapy	18
5.7	Follow-Up	19
5.8	Duration of Study Participation	19
5.9	Supportive Care, Prohibited and Concomitant Therapies/Medications	19
6.0	Dose Modification/ Delay Guidelines	20
7.0	Adverse Events and Unanticipated Problems	26
8.0	Agent Information	34
9.0	Correlative/ Special Studies & Central Review	37
10.0	Study Calendar	40
11.0	Endpoint Definitions/Measurement of Effect	41
12.0	Statistical Considerations	44
12.1	Study Design	44
12.2	Sample Size and Accrual Rate	45
12.3	Statistical Analysis Plan	45
13.0	Protocol Deviations and Single Subject Exceptions	47
14.0	Study Oversight, Quality Assurance, and Data & Safety Monitoring	47
15.0	Ethical and Regulatory Considerations	50
16.0	References	52

Appendix A: ECOG Performance Status 54

LIST OF TABLES AND FIGURES

Table 10.0 Study Activity Calendar 41

ABBREVIATIONS

Abbreviation	Meaning
AE	Adverse Event
CFR	Code of Federal Regulations
COH	City of Hope
CR	Complete Response
CRC	Clinical Research Coordinator
CRF	Case Report Form
CTCAE	Common Terminology Criteria for Adverse Events
DLT	Dose Limiting Toxicity
DSMC	Data & Safety Monitoring Committee
EOT	End of Treatment
FDA	Food and Drug Administration
GCP	Good Clinical Practice
HIV	Human Immunodeficiency Virus
IB	Investigator’s Brochure
IDS	Investigational Drug Services
IND	Investigational New Drug
IRB	Institutional Review Board
NCI	National Cancer Institute
OIDRA	Office of IND Development and Regulatory Affairs
PD	Progressive Disease
PI	Principal Investigator
PMT	Protocol Management Team
PR	Partial Response
SAE	Serious Adverse Event
SD	Stable disease
UP	Unanticipated Problem
ECOG	Eastern Cooperative Oncology Group
RR	Response Rate
PFS	Progression-Free Survival
OS	Overall Survival
OBED	Optimal Biologic Effective Dose
PD-1	Programmed cell Death 1
PD-L1	Programmed cell Death Ligand 1
CTLA-4	Cytotoxic T-Lymphocyte Associated Protein 4
mRCC	Metastatic Renal Cell Carcinoma

1.0 OBJECTIVES AND ENDPOINTS

1.1 Primary Objectives

Objectives	Endpoints/Measurements of Effect
o To determine the effect of CBM588 (in combination with nivolumab/ipilimumab) on the gut microbiome in patients with mRCC	o Primary: Change in *Bifidobacterium* composition of stool from baseline to week 13 of therapy<br>o Secondary: Comparison of the Shannon index (a measure of microbial diversity) from baseline to week 13 of therapy

1.2 Secondary Objective

Objectives	Endpoints/Measurements of Effect
o To evaluate the effect of CBM588 on the clinical efficacy of the nivolumab/ipilimumab combination	o Best overall response, by RECIST criteria, with nivolumab/ipilimumab alone versus nivolumab/ipilimumab with CBM588<br>o Progression-free survival (PFS), assessed as the duration of time from enrollment to progression, with nivolumab/ipilimumab alone vs nivolumab/ipilimumab with CBM588
o To assess the effect of CBM588 on systemic immunodulation of the nivolumab/ipilimumab combination in patients with mRCC	o Comparison of the proportion of circulating Tregs at baseline to levels of circulating Tregs with nivolumab/ipilimumab alone versus nivolumab/ipilimumab with CBM588<br>o Comparison of the proportion of circulating MDSCs with nivolumab/ipilimumab alone versus nivolumab/ipilimumab with CBM588<br>o Comparison of IL-6, IL-8 and other cytokines/chemokines with nivolumab/ipilimumab alone versus nivolumab/ipilimumab with CBM588
o To assess the effect of CBM588 on toxicities such as diarrhea and nausea using CTCAE v5 criteria with the nivolumab/ipilimumab combination in patients with mRCC	o Comparison of toxicities such as diarrhea and nausea using CTCAE v5 criteria with nivolumab/ipilimumab alone versus nivolumab/ipilimumab with CBM588

2.0 BACKGROUND

2.1 Nivolumab/Ipilimumab for mRCC

More than 65,000 patients will be diagnosed with renal cell carcinoma in 2018 in the United States.[1] At the time of initial presentation, one-third of the cases are metastatic, and the remainder have varying rates of progression to metastatic disease. The current treatment algorithm includes surgery followed by a sequence of FDA-approved agents. In the last decade, multiple agents have been approved for treatment of metastatic renal cell carcinoma (mRCC) including targeted therapies (sunitinib, sorafenib, axitinib, pazopanib, everolimus, axitinib, cabozantinib) or immunotherapy with either nivolumab monotherapy or the combination of nivolumab and ipilimumab.[2] Nivolumab and ipilimumab are fully human monoclonal antibodies targeting the programmed cell death protein 1 (PD-1) and the cytotoxic T lymphocyte antigen 4 (CTLA-4) pathway respectively. Inhibition of the PD-1 and CTLA-4 immune checkpoint pathways overcomes the immune escape mechanisms of the tumor cells and allow for enhanced antitumor activity.

In the CheckMate214 trial,[3] the combination of nivolumab and ipilimumab was compared with sunitinib, which was considered the standard of care treatment for patients with mRCC.[4] For patients with intermediate- or poor-risk disease by the International mRCC Database Consortium (IMDC) risk classification, overall survival (OS) and objective response rate (ORR) were significantly improved with the immunotherapy combination versus sunitinib (18-month overall survival 75% vs 60%, objective response rate 42% vs 27%, respectively). However, for patients with favorable-risk disease, the response rate was lower with the combination of nivolumab plus ipilimumab when compared with sunitinib. While these response rates are impressive for intermediate and poor risk disease patients, it is important to note that they reflect the minority of patients with mRCC. Furthermore, approximately 20% of patients who receive the nivolumab/ipilimumab combination in the front-line setting will develop progressive disease. Thus, there are currently significant efforts to build on this regimen and identify novel approaches to improve on the clinical efficacy of the nivolumab/ipilimumab combination.[3]

2.2 The Microbiome in mRCC

In murine models, Vetizou and colleagues have reported that the activity of cytotoxic T-lymphocyte associated protein 4 (CTLA4)-blocking therapies is dependent upon the presence of *Bacteroides spp*.[5] In the context of PD-1, Sivan and colleagues have shown that the clinical activity of anti-PD-1 agents is related to *Bifidobacterium spp*.[6] These preclinical efforts have been bolstered by clinical data presented by Gopalakrishnan and colleagues who reported a correlation between the microbiome composition and response to anti-PD1 agents in 43 patients with metastatic melanoma.[7] Responders to anti-PD-1 agents had significantly higher microbial diversity versus non-responders (P=0.03), and responders higher levels of *Ruminococcacae spp*, respectively (P<0.01).[7] Routy et al conducted a similar analysis across a wider range of epithelial tumors, including 40 patients with mRCC and 60 patients with non-small cell lung cancer (NSCLC). They showed that the relative abundance of *Akkermansia municiphila* was closely linked to response.[8]

Our group has also interrogated the role of the microbiome in mRCC patients receiving nivolumab and TKIs. Patients receiving sunitinib or nivolumab were enrolled through two separate, IRB-approved protocols (COH IRB 16088 and COH IRB 16323, respectively). Sample collection was uniform across the studies, with the first stool specimen collected prior to the onset of treatment, the second collected after 4 weeks of therapy, and the third collected after 12 weeks of therapy. Patients were asked to submit an additional stool sample at the first onset of diarrhea, as well, if this occurred within the 12-week study period. Additional specimens were permitted at the patient's discretion. A manual was given to patients with detailed instructions for stool submission. Briefly, stool was collected at home in a sealed specimen container, surrounded by a cold pack at 4°C, and then placed in a Styrofoam shipping container. Stool was shipped overnight, frozen at -20°C upon receiving, and used for DNA extraction as described below.

Patients were asked to maintain comprehensive food diaries in both studies; however, the two studies differed in dietary restrictions. The first protocol (COH IRB 16088) randomized patients receiving sunitinib to either a diet excluding yogurt or any bacterial fortified foods, or mandated that patients take a standard 4 ounces of a standard yogurt supplement (Activia™) twice daily for the 12-week study period. The second protocol (COH IRB 16323) mandated exclusion of yogurt or bacterial fortified foods for the 12-week study period.

At the end of the 12-week study period, the response was characterized using RECIST 1.1 criteria. For purposes of that study, responders were defined as patients achieving a complete response (CR), partial response (PR) or stable disease (SD) by 3 months of therapy, while non-responders were defined as those with progressive disease (PD) as the best response.

Fecal samples were processed using protocols established by the Earth Microbiome Project (EMP). Briefly, DNA from 250 mg of fecal material for each sample was extracted using the DNeasy PowerSoil kit (MoBio Laboratories, Qiagen Company, Carlsbad, CA). The manufacturer's protocol was followed with the exception of a 10 minute incubation at 65�C after the addition of solution C1 per the EMP protocol. 16S amplicon libraries with barcoded adapters corresponding to the Illumina chemistry were prepared from the extracted DNA using previously described methods. Each library was quantified with qPCR (Kapa Biosystems; Wilmington, MA). The quantified libraries were pooled at equimolar concentrations. The pool was quantified and run on the Illumina MiSeq using version 3 chemistry (Illumina Inc.; San Diego, CA).

Sequence reads were processed by Mothur software, as described in MiSeq SOP, assembled in OUTs, taxonomically annotated to the level of genus and used to construct Bray-Curtis dissimilarity matrix. The similarity of samples was visualized by PCoA and further confirmed by ANOSIM tests, differentially abundant taxa were determined by METASTATS software.

Processed fecal DNA (see methods) was subject to PCR using universal primers. The PCR amplicons were sequenced, rarefied to 10,000 sequences/sample and low-quality sequences were trimmed. Chimeric sequences were removed and assembled in 7,097 operating taxonomic units (OTUs) and taxonomically annotated to the genera level. OTU size ranged from 1 sequence for median and minimal sizes, 37 for average sizes, and 30,878 for maximum sizes. The OTUs were used to assess the structure, membership, and dynamics of the gut microbial community. OTU abundances were standardized and used to calculate distances between samples using Bray-Curtis dissimilarity measure, and visualized by PCoA plot. Distribution of samples confirmed that the structure of gut microbiota was patients specific (ANOSIM, p=0.001) and that the treatment response was among significant factors affecting sample separation (ANOSIM, p=0.01).

The structure of microbial community was compared between patients responding to nivolumab

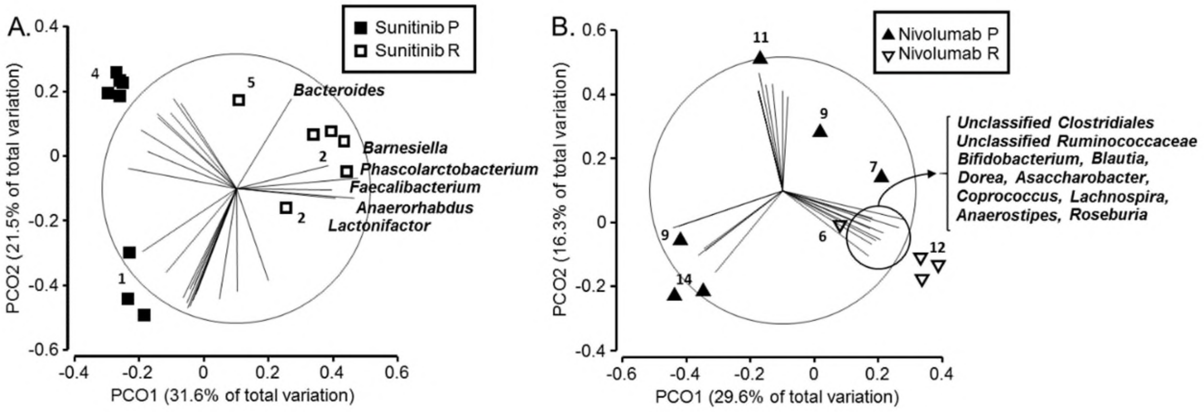

and sunitinib treatment and patients with progressing tumor using 8 samples collected before the initiation of treatment (time point 1 [T1]). The Gini-Simpson index suggested that the complexity of gut microbiota was not significantly different between the response groups (Wilcoxon rank-sum test p=0.25). At the same time, the observed trend suggested that the microbiota of patients responding to the treatment has higher complexity. The structure of gut microbiota was resolved to the level of phylum and genus. METASTATS identified phylum *Bacteroidetes*, genera *Barnesiella*, and *Bacteroides* as elevated among responders (p<0.05 for each). Although phylum *Proteobacteria* was elevated in non-responders, METASTATS analysis suggested that this difference was not significant (p=0.29).

Taken together, our data suggest that specific bacterial species (e.g., *Bifidobacterium, Clostridiales,* etc) are associated with the clinical efficacy associated with immunotherapy in mRCC. We therefore propose the combination of nivolumab/ipilimumab with CBM588 in patients with mRCC as a novel approach of enhancing subpopulations of bacteria, such as *Bifidobacterium.*

2.3 CBM588

CBM588 is a strain of *Clostridium butyricum* isolated from the soil in Nagano, Japan in the 1960s. It has been widely used commercially in Japan as a live biotherapeutic in humans and a feed additive in animals. CBM588 was authorized by the European Union as a novel food ingredient in 2014, and as a feed additive for turkeys, chickens, and related minor avian species.[9] Previous studies have assessed the safety and tolerability of CBM588 in rats or dogs, and no toxicity was observed at dose levels of up to 5000 mg/kg for 12 months.[9] *In vivo* studies of CBM-588 have not identified adverse effects on reproduction or development. Furthermore, these studies showed that the agent promotes restoration of the GI microbiome by stimulating the growth of beneficial intestinal bacteria, including l*actobacilli* and *bifidobacteria*. Butyric acid is known to have immunomodulatory and anti-inflammatory effects on the intestinal epithelium. CBM588 has been demonstrated to significantly reduce ulceration and inflammation in a rat dextran sodium sulfate-induced colitis model. In a pediatric study including 110 children with upper respiratory tract infection or gastroenteritis, CBM588 was safe and well-tolerated. Furthermore, the incidence of antibiotic-related diarrhea was markedly reduced in patients who received CBM588 (59% vs 5%).[10] In a study of ulcerative colitis, CBM588 was administered at a dose of 60 mg oral tid.[11,12]

Although the agent is commercially available in Japan, there is limited data to document the safety and tolerability of the combination of CBM588 with immunotherapy in patients with advanced cancer. We propose assessing CBM588 as an adjunct to nivolumab/ipilimumab in patients with mRCC in a phase I protocol. The OBED will represent the dose that results in the greatest increase in *Bifidobacterium* from baseline to week 12 of therapy. *Bifidobacterium* is selected amongst other putative bacteria that are associated with response to immunotherapy, since it is hypothesized that CBM588 will specifically increase levels of this genus.

3.0 ELIGIBILITY CRITERIA

Participants must meet all of the following criteria on screening examination to be eligible to participate in the study.

3.1 Inclusion Criteria

3.1.1 Be willing and able to provide informed consent for the trial.

3.1.2 Histological confirmation of RCC with a clear-cell or sarcomatoid component.

3.1.3 Advanced (not amenable to curative surgery or radiation therapy) or metastatic (AJCC Stage IV) RCC.

3.1.4 Intermediate or poor risk disease by IMDC classification

3.1.5 No prior systemic therapy for RCC with the following exception:

3.1.5.1 One prior adjuvant or neoadjuvant therapy for completely resectable RCC if such therapy did not include an agent that targets PD-1 or PD-L1 and if recurrence occurred at least 6 months after the last dose of adjuvant or neoadjuvant therapy.

3.1.6 ECOG Performance Status < 2 (See Appendix A).

3.1.7 Measurable disease as per RECIST 1.1.

3.1.8 Males and females, ages ≥18.

3.1.9 Any ethnicity or race.

3.1.10 Adequate renal function defined as calculated creatinine clearance ≥30 milliliters per minute (mL/min) per the Cockcroft and Gault formula or Serum creatinine < 1.5 x upper limit of normal (ULN)

3.1.11 Adequate liver function defined by AST or ALT < 3 x ULN (< 5 x ULN if liver metastases are present), and total bilirubin < 1.5 x ULN (except subjects with Gilbert Syndrome, who can have total bilirubin up to 3.0 mg/dL)

3.1.12 Adequate bone marrow function defined by any of the following laboratory test findings: WBC > 2,000/mm3, Neutrophils > 1,500/mm3, Platelets > 100,000/mm3

3.2 Key Exclusion Criteria:

3.2.1 Presence of untreated brain metastases. Patients with treated brain metastases must be stable for 4 weeks after completion of treatment and have documented stability on pre-study imaging. Patients must have no clinical symptoms from brain metastases and have no requirement for systemic corticosteroids amounting to >10 mg/day of prednisone or its equivalent for at least 2

weeks prior to first dose of study drug. Patients with known leptomeningeal metastases are excluded, even if treated.

3.2.2 Favorable risk disease by IMDC classification.

3.2.3 Prior treatment with an anti-PD-1, anti-PD-L1, anti-PD-L2, anti-CD137, or anti-CTLA-4 antibody, or any other antibody or drug specifically targeting T-cell co-stimulation or checkpoint pathways.

3.2.4 Any active or recent history of a known or suspected autoimmune disease or recent history of a syndrome that required systemic corticosteroids (> 10 mg daily prednisone equivalent) or immunosuppressive medications except for syndromes which would not be expected to recur in the absence of an external trigger. Subjects with vitiligo or type I diabetes mellitus or residual hypothyroidism due to autoimmune thyroiditis only requiring hormone replacement are permitted to enroll.

3.2.5 Active interstitial lung disease (ILD)/pneumonitis or history of ILD/pneumonitis requiring treatment with systemic steroids

3.2.6 Baseline pulse oximetry less than 92% "on Room air"

3.2.7 Current use, or intent to use, probiotics, yogurt or bacterial fortified foods during the period of treatment.

3.2.8 Any condition requiring systemic treatment with corticosteroids (> 10 mg daily prednisone equivalents) or other immunosuppressive medications within 14 days prior to first dose of study drug. Inhaled steroids and adrenal replacement steroid doses > 10 mg daily prednisone equivalents are permitted in the absence of active autoimmune disease.

3.2.9 Uncontrolled adrenal insufficiency.

3.2.10 Known medical condition (e.g., a condition associated with diarrhea or acute diverticulitis) that, in the investigator's opinion, would increase the risk associated with study participation or study drug administration or interfere with the interpretation of safety results.

3.2.11 Not recovered to ≤ Grade 1 toxicities related to any prior therapy before administration of study drug..

3.2.12 Women who are pregnant or breastfeeding.

3.2.13 History of myocarditis or congestive heart failure (as defined by New York Heart Association Functional Classification III or IV), as well as unstable angina, serious uncontrolled cardiac arrhythmia, uncontrolled infection, or myocardial infarction 6 months prior to study entry

3.2.14 Any of the following laboratory test findings:

3.2.14.1 WBC < 2,000/mm^3

3.2.14.2 Neutrophils < 1,500/mm^3

3.2.14.3 Platelets < 100,000/mm^3

3.2.14.4 AST or ALT > 3 x ULN (> 5 x ULN if liver metastases are present)

3.2.14.5 Total bilirubin > 1.5 x ULN (except subjects with Gilbert Syndrome, who can have total bilirubin 3.0 mg/dL)

3.2.14.6 Calculated creatinine clearance <30 millimeters per minute (mL/min) per the Cockcroft and Gault formula or serum creatinine >1.5 x upper limit of normal (ULN)

4.0 PARTICIPANT RECRUITMENT, ENROLLMENT

This trial will be conducted as a single-center site in the City of Hope Comprehensive Cancer Center.

Eligible patients will be enrolled on study at the City of Hope Comprehensive Cancer Center by the Study Coordinator.

To register a patient, the eligibility packet should be completed by the research nurse/coordinator and sent to the data coordinating center (DCC) at DL-dcc@coh.org for review. DCC requires a 24-hour window for review of complete eligibility packets. Once complete, DCC will confirm eligibility and provide randomization to study arm, along with a confirmation of registration form and DCC signed checklist.

5.0 TREATMENT PROGRAM

5.1 Treatment Program Overview

This is a Phase I study and will consist of a randomization to one of 2 treatment arms to ascertain the biologic effect of nivolumab/ipilimumab with CBM588. Please refer to Statistical Analysis for details of randomization. Dose levels for each treatment arm are noted below:

Treatment Arms

Dose Level	Sample Size	Dose		
		**CBM588 All Cycles**	**Nivolumab/Ipilimumab Cycles 1-4**	**Nivolumab Cycles 5 +**
**Arm 1**	**10**	None	3 mg/kg / 1 mg/kg	480 mg
**Arm 2**	**20**	80 mg bid	3 mg/kg / 1 mg/kg	480 mg

5.2 Cycle Definition

One cycle of therapy will constitute an every-3-week regimen of nivolumab with ipilimumab for the first 4 cycles (12 weeks). Thereafter, a cycle will be considered 4 weeks, as nivolumab will be administered on a monthly schedule thereafter.

5.3 Treatment Plan

CBM588:

CBM588 will be administered orally on a daily basis, according to the allocated treatment arm per Section 5.1. Each dose to be taken twice daily (BID; am/pm), and at the same time each day or within a few hours of the regular time. Missed doses are allowed and should be documented on the study drug log. Emergency medications are per standard of care.

Nivolumab:

Nivolumab will be administered at a dose of 3 mg/kg intravenously prior to administration of ipilimumab for the first 4 cycles followed by monthly dosing at 480 mg. Emergency medications are per standard of care.

Ipilimumab:

Ipilimumab will be administered after nivolumab administration, at a dose of 1 mg/kg intravenously for only the first 4 cycles, after which point, it will be discontinued. Emergency medications are per standard of care.

5.4 Agent Administration

CBM588:

CBM588 will be supplied by Osel, Inc. CBM588 Fine Granules are manufactured by Mirayasan as an orally available live biotherapeutic comprised of *Clostridium butyricum* and packaged in 1 g sachets. Each sachet contains 40 mg of CBM588. CBM588 will be administered orally at a dose of 80 mg twice daily in 100 ml of water (the contents of two sachets) and should be given indefinitely while on protocol. Subjects can take CBM588 with or without food. CBM588 should be taken at home, not in clinic. The participants will be requested to maintain a study log, which includes a diet and stool frequency log, as well as a medication diary of each dose of medication. The study log will be returned to clinic staff at the end of each course.

Nivolumab

Nivolumab will be supplied by City of Hope Comprehensive Cancer Center and billed to patients and/or their third-party payer. Nivolumab injection is a clear opalescent, colorless to pale yellow, sterile, non-pyrogenic, single-use, isotonic aqueous solution formulated in sodium citrate, sodium chloride, mannitol, diethylenetriamine pentacetic acid (pentetic acid) and polysorbate 80 (Tween® 80), pH 6.0. Each vial is 100 mg (10 mg/mL) with a 0.7 mL overfill in 10 mL type I flint glass vials, with butyl rubber stoppers and aluminum seals. Vials of Nivolumab injection must be stored at 2°-8° C (36°F-46°F) and protected from light, freezing and shaking. If a storage temperature excursion is identified, promptly return Nivolumab to 2°C to 8°C and quarantine the supplies.

Nivolumab can be infused undiluted (10 mg/mL) or diluted with 0.9% Sodium Chloride injection, USP or 5% dextrose, USP to drug concentrations no less than 0.35 mg/mL. Note: mix gently and do not shake. Do **NOT** administer as IV push or bolus injection. Nivolumab injection is to be administered as a 30 minute

IV infusion through a 0.2 micron to 1.2 micron pore size, low protein binding polyethersulfone membrane in-line filter. No compatibilities between nivolumab and polyvinyl chloride (PVC), nonPVC DHEP (di(2-ethylhexyl)phthalate) IV components, or glass bottles have been observed.

The administration of undiluted and diluted solutions of nivolumab must be completed within 24 hours preparation. If not used immediately, the infusion solution may be stored up to 24 hours in a refrigerator at 2°C-8°C (36°F-46°F) and a maximum of 4 hours of the total 24 hours can be at room temperature (20°C-25°C (68°F-77°F)) and room light. The maximum 4-hour period under room temperature and room light conditions includes the product administration period.

CAUTION: The single-use dosage form contains no antibacterial preservative or bacteriostatic agent. Therefore, it is advised that the product be discarded 8 hours after initial entry.

Ipilimumab

Ipilimumab will be supplied by City of Hope Comprehensive Cancer Center and billed to patients and/or their third-party payer. Ipilimumab injection is supplied as 200 mg/40 mL (5 mg/mL). It is formulated as a clear to slightly opalescent, colorless to pale yellow, sterile, non-pyrogenic, single-use, isotonic aqueous solution that may contain particles. Vials of ipilimumab injection must be stored at 2°C-8°C (36°F-46°F) and protected from light, freezing. If a storage temperature excursion is identified, promptly return ipilimumab to 2°C to 8°C and quarantine the supplies.

Ipilimumab is given undiluted (10 mg/mL) or further diluted in 0.9% Sodium Chloride injection, USP or 5% dextrose, USP in concentrations between 1 mg/mL and 4 mg/mL. Ipilimumab is stable in polyvinyl chloride (PVC), nonPVC DHEP (di(2-ethylhexyl)phthalate) IV bag or glass container up to 24 hours refrigerate at 2°C-8°C (36°F-46°F) or at room temperature/room light. The product may be infused using a volumetric pump at the protocol specific dose(s), nonpyrogenic, low-protein-binding filter (pore size of 0.2 micrometer or 1.2 micrometer). DO NOT administer as IV push or bolus injection.

Prepared IV ipilimumab solution is stable up to 24 hours refrigerated at 2°-8°C (36°F-46°F) or at room temperature/room light. Each vial is a type I flint glass vial with gray butyl stoppers and sealed with aluminum seals.

Partially used vials or empty vials of ipilimumab injection should be discarded at the site according to appropriate drug disposal procedures.

**Regimen Description**					
**Agent**	**Premedications; Precautions**	**Dose**	**Route**	**Schedule**	**Cycle Length**
CBM588	No prophylactic medication should be given.	80 mg	Oral	Twice per day indefinitely while on protocol.	21 days (3 weeks) for cycles 1-4<br><br>28 days (4 weeks) for cycles 5+
Nivolumab	No prophylactic medication should be given	3 mg/kg (for cycles 1-4)	30 minutes IV infusion	Day 1	21 days (3 weeks) for cycles 1-4

	unless indicated by previous indication.	480 mg (for cycles 5+)			28 days (4 weeks) for cycles 5+
Ipilimumab	No prophylactic medication should be given unless indicated by previous indication.	1 mg/kg	30 minutes IV infusion	Day 1	21 days (3 weeks) for cycles 1-4, then discontinued

5.5 Assessments and Special Monitoring

For a detailed list of all study procedures including timing and windows, see Section 10.0.

Patients will be seen on day 1 of each cycle for toxicity assessment. For the purpose of this study, patients should be re-evaluated for response every 12 weeks. Response assessments include CT scan of the chest, abdomen, and pelvis and (if baseline bone disease is documented) bone scan. PET-CT or MRI may be substituted for CT if necessary, but evaluation with the imaging modality used at baseline is preferred.

Participants will be oriented to complete the daily study log, found in **Appendix C**. The log will be collected and reviewed during visits to the clinic at the end of each visit. The following information from the diary will be entered into the study specific database for analysis:

- Diarrhea, categorized per CTCAE 5.0 diarrhea grading. Please note that the patient will be instructed to consider diarrhea to be an increase in stool frequency of < 4 over baseline. This definition is consistent with the CTCAE 5.0 terminology for grade 1 diarrhea.
- Stool specimen collection dates
- Yogurt intake, or intake of yogurt-containing foods
- Medication diary

5.6 Duration of Therapy and Criteria for Removal from Protocol Therapy

Participants will receive protocol therapy until one of the below criteria are met:

- Disease progression
- Completed protocol therapy
- Participant is deemed intolerant to protocol therapy because of toxicity, despite dose modification/ delay
  - **Note:** If one agent is discontinued due to toxicity, then the participant may continue to receive the other study agents
- General or specific changes in the patient's condition which render the patient unacceptable for further treatment in the judgment of the investigator
- Withdrawal of consent for further protocol therapy
- Once participants meet criteria for removal from protocol therapy, the participant should then proceed to End of Treatment assessments, and then to follow-up.

Documentation of the reason for discontinuing protocol therapy and the date effective should be made in the Electronic Health Record/medical record and appropriate eCRF. The COH DCC and the Study PI should be promptly notified of the change in participant status.

5.7 Follow-Up

All participants will enter follow-up after completing End of Treatment assessments. This is comprised of:

- **Safety Follow-up**- 30 days post-last dose of protocol therapy.
  - **Note** the period for safety follow-up will be extended until stabilization or resolution for all reportable AEs (per the agreement of the Study PI) and accompanying follow-up safety report.
- **Response Follow-up**- for those who have yet to have disease progression.
- **Survival Follow-up**- for all participants who have progressed OR completed Active Response Follow-Up.

Assessment time points and windows are detailed in Section 10.0.

5.8 Duration of Study Participation

Study participation may conclude when any of the following occur:

- Completion of study activities
- Withdrawal of consent
- Participant is lost to follow-up. All attempts to contact the participant must be documented.
- At the discretion of the investigator for safety, behavioral, study termination or administrative reasons

Documentation of the reason for discontinuing study participation and the date effective should be made in the Electronic Health Record/medical record and appropriate eCRF. The COH DCC should be promptly notified of the change in participant status.

5.9 Supportive Care, Prohibited and Concomitant Therapies/Medications

If concomitant therapy must be added or changed, including over-the-counter medications or alternative therapies, the reason and name of the agent/therapy should be recorded in the eCRF and documented in the Electronic Health Record/medical record.

5.9.1 Prohibited therapies

Patients are asked not to take any other probiotic agents while on the current protocol. Steroid therapy refers to continuous use rather than tapered treatment.

Given use of nivolumab/ipilimumab for augmentation of antitumor immunity, the following medications are prohibited during the study:

- Immunosuppressive agents (except to treat a drug-related adverse event)
- Systemic corticosteroids > 10 mg daily prednisone equivalent (except to treat a drug-related adverse event).
- Any concurrent antineoplastic therapy (ie, chemotherapy, hormonal therapy, immunotherapy, radiation therapy (except for the purpose of palliation), surgical resection (except for the purpose of palliation)

5.9.2 Supportive care

With the exception of prohibited therapies, participants should receive prophylactic or supportive as clinically indicated per institutional policies.

6.0 DOSE MODIFICATION/ DELAY GUIDELINES

6.1 CBM588

If treatment with nivolumab and/or ipilimumab is deferred, CBM588 should continue irrespective of dose delays or holds to nivolumab/ipilimumab. Dose modification or delays will not be permitted. If clinically significant non-hematologic adverse events occur (grade 3 or above, or persistent grade 2 toxicity at the investigators discretion) that are attributed to CBM588, then the agent should be discontinued.

6.2 Nivolumab in combination with ipilimumab or maintenance therapy

The allowed treatment window for administration of nivolumab and ipilimumab is +/- 7 days from Day 1 of the current cycle. Dose modifications for nivolumab in combination with ipilimumab by adverse event type are consistent with nivolumab and ipilimumab package insert. General considerations for dose modifications are as follows.

For the initial induction period, patients with Grade 2 or 3 events requiring discontinuation of treatment with the combination may consider continuing treatment with single agent nivolumab when the event resolves to baseline. However, patients with renal, CNS, or pulmonary toxicity must be removed from study.

- In addition to the adverse events identified in the table below, ipilimumab and nivolumab dose should be delayed for any adverse event, laboratory abnormality or inter-current illness which, in the judgment of the treating investigator, warrants delaying the dose of study medication
- Patients requiring a delay of >12 weeks or who experience immune-related toxicity with inability to decrease prednisone ≤10mg per oral daily must go off protocol therapy entirely.
- Patients who received systemic corticosteroids for continuous management of any drug-related immunologic toxicity must be off protocol.
- Nivolumab monotherapy may be continued at treating investigator discretion of there is evidence of clinical benefit. In this event, patient will continue nivolumab until AE is resolved and ipilimumab may be re-started or if ipilimumab is permanently discontinued (incongruence with guidelines in table below), then patient will continue nivolumab alone until cycles are completed. At completion of 4 cycles, patient may continue on nivolumab alone, as per protocol.
- If a patient experiences several adverse events and there are conflicting recommendations, the investigator should use the recommended dose adjustment that reduces the dose to the lowest level.

Non-hematologic adverse events

<u>Nausea</u>	**Management/Next Dose for Nivolumab and/or Ipilimumab**
≤ Grade 1	No change in dose
Grade 2	Hold until ≤ Grade 1 or same as baseline (exceptions as noted below)*
Grade 3	Off protocol therapy (exceptions as noted below)
Grade 4	Off protocol therapy
*Patients requiring a delay of >2 weeks should go off protocol therapy.	
Recommended management: antiemetics.	

<u>Diarrhea</u><br><u>Immune-related colitis</u>	**Management/Next Dose for Nivolumab and/or Ipilimumab**
≤ Grade 1	No change in dose
Grade 2	Hold until ≤ Grade 1 or same as baseline (exceptions as noted below)*
Grade 3	Off protocol therapy (exceptions as noted below)
Grade 4	Off protocol therapy
*Patients requiring a delay of >2 weeks should go off protocol therapy.	
Recommended management: Loperamide antidiarrheal therapy<br>Dosage schedule: 4 mg at first onset, followed by 12 mg with each loose motion until diarrhea-free for 12 hours (maximum dosage: 16mg/24 hours)<br>Adjunct anti-diarrheal therapy is permitted and should be recorded when used. See GI adverse event algorithm for management of systemic colitis.<br>Patients with grade 2 symptoms but normal colonoscopy and biopsies may be retreated after resolution.<br>Patients who require steroids must be taken off study treatment. Please evaluate pituitary function prior to starting steroids if possible without compromising acute care. Evaluation for all patients for additional causes may include C. Diff, acute and self-limited infection, and food-borne illness, ischemic bowel, diverticulitis and IBD.	

Vomiting	Management/Next Dose for<br>Nivolumab and/or ipilimumab
≤ Grade 1	No change in dose
Grade 2	Hold until ≤ Grade 1 or same as baseline (exceptions as noted below)*
Grade 3	Off protocol therapy (exceptions as noted below)
Grade 4	Off protocol therapy
*Patients requiring a delay of >2 weeks should go off protocol therapy.	
Recommended management: antiemetics.	

Other GI	Management/Next Dose for<br>Nivolumab and/or ipilimumab
≤ Grade 1	No change in dose
Grade 2	Hold until ≤ Grade 1 or same as baseline (exceptions as noted below)*
Grade 3	Off protocol therapy (exceptions as noted below)
Grade 4	Off protocol therapy
*Patients requiring a delay of >2 weeks should go off protocol therapy.	
Patients with grade 2-3 nausea and vomiting should be evaluated for upper GI inflammation and other immune-related events.	

Neurologic events (new, motor, sensory, encephalitis)	Management/Next Dose for<br>Nivolumab and/or Ipilimumab
≤ Grade 1	No change in dose
Grade 2	Hold until ≤ Grade 1 or same as baseline (exceptions as noted below)*
Grade 3	Off protocol therapy (exceptions as noted below)
Grade 4	Off protocol therapy

Fatigue	Management/Next Dose for Nivolumab and/or Ipilimumab
≤ Grade 1	No change in dose
Grade 2	No change in dose
Grade 3	Off protocol therapy (exceptions as noted below) If fatigue is found related to endocrinopathy, and clinical symptoms are managed with hormone replacement, patient resume therapy.
Grade 4	Off protocol therapy
Fatigue is the most common adverse event associated with immune checkpoint inhibitor therapy. Grade 2 or greater fatigue should be evaluated associated underlying organ involvement including pituitary, thyroid, hepatic, or muscle (CPK) inflammation.	
Recommended management: antiemetics.	

Fever	Management/Next Dose for Nivolumab and/or Ipilimumab
≤ Grade 1	Evaluate and continue
Grade 2	Hold until ≤ Grade 1
Grade 3	Hold until ≤ Grade 1
Grade 4	Off protocol therapy
Patients with fever should be evaluated as clinically appropriate, patient may experience isolated fever during infusion reactions or up to several days after infusion. Evaluation over the course of 1 to 2 weeks should be done for other autoimmune events that may present with fever.	

Skin drug-related adverse events	Management/Next Dose for Nivolumab and/or Ipilimumab
≤ Grade 1	No change in dose
Grade 2	Hold until ≤ Grade 1 or same as baseline (exceptions as noted below)*
Grade 3	Off protocol therapy (exceptions as noted below)
Grade 4	Off protocol therapy
Patients wth purpuric or bullous lesions must be evaluated for vasculitis, Steven Johnsons syndrome, TEN, and autoimmune bullous disease including oral lesions of bullous	

Skin drug-related adverse events	Management/Next Dose for Nivolumab and/or Ipilimumab
pemphigus/pemphigoid. Pruritus may occur with or without skin rash and should be treated symptomatically if there is no associated liver of GI toxicity. Note skin rash typically occurs early and may be followed by additional events particularly during steroid tapering.	

Pneumonitis, bronco-spasm, pulmonary toxicity or intestinal lung disease	Management/Next Dose for Nivolumab and/or Ipilimumab
≤ Grade 1	No change in dose,
Grade 2	Hold dose pending evaluation. Resume after pulmonary and/or ID consultation excludes autoimmune related causes. Patient must be removed from protocol therapy if steroids are required.
Grade 3	Off protocol therapy
Grade 4	Off protocol therapy
	Above does not include infusion reactions
Distinguishing inflammatory pneumonitis is often a diagnosis of exclusion for patients who do not respond to antibiotics and have no casual organisms identified including influenza. Most patients with respiratory failure or hypoxia will be treated with steroids. Bronchoscopy may be required and analysis of lavage fluid for lymphocytic predominance may be helpful. Patients with new lung nodules should be evaluated for sarcoid line granuloma. Please consider recommending seasonal influenza killed vaccine for all patients.	

Renal	Management/Next Dose for Nivolumab and/or Ipilimumab
≤ Grade 1	No change in dose
Grade 2	Hold until ≤ Grade
Grade 3	Off protocol therapy
Grade 4	Off protocol therapy

Endocrine<br>Hypophysitis<br>Adrenal Insufficiency	Management/Next Dose for<br>Nivolumab and/or Ipilimumab
≤ Grade 1	No change in dose
Grade 2	Hold until patients are on a stable replacement hormone regimen. If treated with steroids, patients must be stable off steroids for two weeks.
Grade 3	Hold until patients are on a stable replacement hormone regimen. If treated with steroids, patients must be stable off steroids for two weeks.
Grade 4	Off protocol therapy
Note al patients with symptomatic pituitary enlargement, exclusive of hormone deficiency, but including severe headache or enlarged pituitary on MRI should be considered as Grade 3 events. Isolated thyroid or testosterone deficiency may be treated as Grade 2 if there are no other associated deficiencies and adrenal function is monitored, Please evaluate pituitary function before beginning steroid therapy or replacement therapy of any kind.<br>Note patients with thyroiditis may be retreated on replacement therapy. Patients must be evaluated to rule out pituitary disease prior to initiating thyroid replacement.	

Hematologic adverse events

Thrombocytopenia	Management/Next Dose for<br>Nivolumab and/or ipilimumab
≤ Grade 1	No change in dose
Grade 2	Hold until ≤ Grade 1 or baseline. (exceptions as noted below)
Grade 3	Off protocol therapy
Grade 4	Off protocol therapy
*Patients requiring a delay of >2 weeks should go off protocol therapy.	

Neutroopenia	Management/Next Dose for<br>Nivolumab and/or ipilimumab
≤ Grade 1	No change in dose

Neutroopenia	Management/Next Dose for Nivolumab and/or ipilimumab
Grade 2	Hold until ≤ Grade 1 or baseline. (exceptions as noted below)
Grade 3	Off protocol therapy
Grade 4	Off protocol therapy
*Patients requiring a delay of >2 weeks should go off protocol therapy.	

6.3 Early Stopping

After the enrollment of the first 10 patients, the study will be paused for a 30 day window. If the rate of treatment-related grade 3/4 events exceeds 60% or if any treatment-related deaths have occurred, enrollment to the study will be halted pending further discussion with the FDA. The threshold of 60% was based on a 46% incidence of grade 3/4 events noted in the CheckMate214 study.

7.0 ADVERSE EVENTS AND UNANTICIPATED PROBLEMS

7.1 Definitions

7.1.1 Adverse Event (AE)

An adverse event is any untoward medical experience or change of an existing condition that occurs during or after treatment, whether or not it is considered to be related to the protocol intervention.

7.1.2 Serious Adverse Event (SAE)

A serious adverse event is any expected or unexpected adverse events that result in any of the following outcomes:

- Death
- Is life-threatening experience (places the subject at immediate risk of death from the event as it occurred)
- Unplanned hospitalization (equal to or greater than 24 hours) or prolongation of existing hospitalization
- A persistent or significant disability/incapacity
- A congenital anomaly/birth defect
- Secondary malignancy*
- Any other adverse event that, based upon appropriate medical judgment, may jeopardize the subject's health and may require medical or surgical intervention to prevent one of the outcomes listed above (examples of such events include allergic bronchospasm requiring intensive treatment in the emergency room or at home, blood dyscrasias of convulsions that do not result in inpatient hospitalization, or the development of drug dependency or drug abuse).

*Modified from 21 CFR 312.32

7.1.3 Unanticipated Problems Involving Risks to Subjects or Others

An unanticipated problem is any incident, experience, or outcome that **meets all three** of the following criteria:

1. Unexpected (in terms of nature, severity, or frequency) given the following: a) the research procedures described in the protocol-related documents such as the IRB approved research protocol, informed consent document or Investigator Brochure (IB); and b) the characteristics of the subject population being studied; **AND**
2. Related or possibly related to participation in the research (possibly related means there is a reasonable possibility that the incident, experience, or outcomes may have been caused by the drugs, devices or procedures involved in the research); **AND**
3. Suggests that the research places subjects or others at greater risk of harm (including physical, psychological, economic, or social harm) than previously known or recognized.

7.1.4 Adverse Events of Special Interest (AESI)

Specific adverse events, or groups of adverse events, will be followed as part of standard safety monitoring activities. These events, regardless of seriousness, will be reported.

7.1.4.1 Study specific AESIs

- **CBM588**

To date, clinical studies of CBM588 have not reported any adverse events. The potential side effects with probiotic therapies include abdominal pain, constipation, diarrhea, and fatigue.

- **Nivolumab with Ipilimumab**

With dual checkpoint therapy, it is challenging to ascribe toxicity to either agent alone – as such, the collective side effects potentially associated with this regimen are noted below:

Expected (known) toxicities to nivolumab with ipilimumab

Adverse Events with Possible Relationship to Anti-CTLA4 immunotherapy (CTCAE v5.0 Term) (n=2069)			Specific Protocol Exceptions to Expedited Reporting (SPEER)
Likely (>20%)	Less likely (≤20%)	Rare but serious (<3%)	
BLOOD AND LYMPHATIC SYSTEM DISORDERS			
		Blood and lymphatic system disorders – Other (acquires hemophilia)	
CARDIAC DISORDERS			
	Atrial fibrillation		
		Myocarditis[2]	
EAR AND LABYRINTH DISORDERS			
	Hearing impaired		
ENDOCRINE DISORDERS			
	Adrenal insufficient[2]		
	Endocrine disorders – Other (hypopituitarism/hypophysitis)[2]		
	Endocrine disorders – Other (testosterone deficiency)[2]		
	Hyperthyroidism[2]		
	Hypothyroidism[2]		
EYE DISORDER			
	Eye disorders – Other (Episcleritis)[2]		
	Uveitis[2]		
GASTROINTESTINAL DISORDERS			
	Abdominal pain		Colitis (Gr 3)
	Colitis[2]		
		Colonic perforation	
	Constipation		
Diarrhea			Diarrhea (Gr 3)
	Enterocolitis		
	Esophagia		
		Ileus	
Nausea			Nausea (Gr 3)
	Pancreatitis[2]		
	Vomiting		
GENERAL DISORDERS AND ADMINISTRATION SITE CONDITION			
	Chills		

Fatigue			Fatigue (Gr 3)
	Fever		Fever (Gr 2)
	Infusion related reaction		
		Multi-organ failure	
HEPATOBILIARY DISORDERS			
	Hepatobiliary disorders – Other (hepatitis)[2]		
IMMUNE SYSTEM DISORDERS			
	Autoimmune disorder[2]		
		Immune system disorders 0 Other (GVHD in setting of allotransplant)[4]	
INFECTIONS AND INFESTATIONS			
		Infections and infestations - Other (aseptic meningitis)	
INVESTIGATIONS			
	Alanine aminotransferase increased		
	Aspartate aminotransferase increased		
	Neutrophil count decreased		
METABOLISM AND NUTRITION DISORDERS			
	Anorexia		
	Dehydration		
	Hyperglycemia		
		Metabolism and nutrition disorders – Other (exacerbation of pre-existing diabetes mellitus)	
MUSCULOSKELETAL AND CONNECTIVE TISSUE DISORDERS			
	Arthralgia		
	Arthritis		
	Musculoskeletal and connective tissue disorders – Other (polymyositis)[2]		
NERVOUS SYSTEM DISORDERS			
	Facial nerve disorder		

	Headache		
	Nervous system disorder – Other (Guillain-Barre syndrome)[2]		
	Nervous system disorder – Other (myasthenia gravis)[2]		
	Trigeminal nerve disorder		
RENAL AND URINARY DISORDERS			
	Acute kidney injury		
	Renal and urinary disorders – Other (granulomatous tubulointerstitial nephritis)		
RESPIRATORY, THORACIC AND MEDIASTINAL DISORDERS			
	Pneumonitis		
		Respiratory, thoracic and mediastinal disorders – Other (bronchiolitis obliterans with organisin pneumonia)	
SKIN AND SUBCUTANEOUS TISSUE DISORDERS			
		Erythema multiforme	
	Pruritus		**Pruritus (Gr 3)**
Rash maculo-papular			**Rash maculo-papular (Gr 3)**
	Skin and cutaneous disorders – other (Sweet's syndrome)		
		Stevens-Johnson syndrome	
		Toxic-epidermal necrolysis	
	Urticaria		
VASCULAR DISORDERS			
	Hypotension		

7.2 Assessment of Adverse Events

The site Investigator will be responsible for determining the event name, assessing the severity (i.e. grade), expectedness, and attribution of all adverse events.

7.2.1 Assessment of Adverse Event Name and Grade

Adverse events will be characterized using the descriptions and grading scales found in the most recent version of Common Terminology Criteria for Adverse Events (CTCAE) v5.0. A copy of the scale can be

found at https://ctep.cancer.gov/protocolDevelopment/electronic_applications/ctc.htm#ctc_50. The determination of severity for all other events not listed in the CTCAE v5.0 should be made by the investigator based on medical judgment and the severity categories of Grade 1 to 5 as defined below:

- Grade 1 (mild) – An event that is usually transient and may require only minimal treatment or therapeutic intervention. The event does not generally interfere with usual activities of daily living.
- Grade 2 (moderate) – An event that is usually alleviated with additional specific therapeutic intervention. The event interferes with usual activities of daily living, causing discomfort but poses no significant or permanent risk of harm to the subject.
- Grade 3 (severe) – An event that requires intensive therapeutic intervention. The event interrupts usual activities of daily living, or significantly affects the clinical status of the subject.
- Grade 4 (life threatening) – An event, and/or its immediate sequelae, that is associated with an imminent risk of death or with physical or mental disabilities that affect or limit the ability of the subject to perform activities of daily living (eating, ambulation, toileting, etc).
- Grade 5 (fatal) – Death (loss of life) as a result of an event.

7.2.2 Assessment of Attribution

The following definitions will be used to determine the causality (attribution) of the event to the study agent or study procedure.

- **Unrelated –** The event is clearly related to other factors such as the participant's clinical state, other therapeutic interventions, or concomitant medications administered to the participant.
- **Unlikely –** The event is doubtfully related to the investigational agent(s). The event was most likely related to other factors such as the participant's clinical state, other therapeutic interventions, or concomitant drugs.
- **Possible –** The event follows a reasonable temporal sequence from the time of drug administration, but could have been produced by other factors such as the participant's clinical state, other therapeutic interventions, or concomitant drugs.
- **Probable –** The event follows a reasonable temporal sequence from the time of drug administration, and follows a known response pattern to the study drug. The event cannot be reasonably explained by other factors such as the participant's clinical state, therapeutic interventions, or concomitant drugs.
- **Definite –** The event follows a reasonable temporal sequence from the time of drug administration, follows a known response pattern to the study drug, cannot be reasonably explained by other factors such as the participant's condition, therapeutic interventions, or concomitant drugs, AND occurs immediately following study drug administration, improves upon stopping the drug, or reappears on re-exposure.

7.2.3 Assessment of Expectedness

The following definitions will be used to determine the expectedness of the event:

- **Unexpected** – An adverse event is unexpected if it is not listed in the investigator's brochure and/or package insert; is not listed at the specificity or severity that has been observed; is not consistent with the risk information described in the protocol and/or consent; is not an expected natural progression of any underlying disease, disorder, condition, or predisposed risk factor of the research participant experiencing the adverse event. *Modified from 21 CFR 312.32 (a)
- **Expected –** An adverse event is expected if it does not meet the criteria for an unexpected event, OR is an expected natural progression of any underlying disease, disorder, condition, or predisposed risk factor of the research participant experiencing the adverse event.

7.3 Reporting of Adverse Events

7.3.1 Routine Reporting of Non-Serious Adverse Events

Routine AE recording will occur via data entry into the study eCRF. Recording of adverse events will begin once the patient is consented and will continue until the patient completes, discontinues, or withdraws from the study. Adverse events will be monitored by the Protocol Management Team (PMT). Adverse events that do not meet the criteria of serious OR are not unanticipated problems do not require expedited reporting. AEs reported through expedited processes (i.e. reported to the IRB, DSMC, FDA, etc.) must also be reported in routine study data submissions.

7.3.2 Expediting Reporting Requirements of SAEs and UPs

Adverse events that meet the criteria of serious OR are unanticipated problems will be reported according to the approved City of Hope's Institutional policy via the AE/UP reporting form in iRIS. Reportable serious adverse events must be followed until the event is resolved, stabilized, or determined to be irreversible by the investigator. Follow-up SAE reports must be submitted for all events that require expedited reporting when the status of the event changes and until the resolution or stabilization of the event.

7.3.3 Additional AE Reporting Requirements

7.3.3.1 Reporting to the FDA

The study PI (or designee) will be responsible for contacting the Office of IND Development and Regulatory Affairs (OIDRA) at COH to ensure prompt reporting of safety reports to the FDA. OIDRA will assist the PI with the preparation of the report and submit the report to the FDA in accordance with the approved City of Hope's Institutional policy..

Serious Adverse Events meeting the requirements for expedited reporting to the Food and Drug Administration (FDA), as defined in 21 CFR 312.32, will be reported as an IND safety report using the MedWatch Form FDA 3500A for Mandatory Reporting.

The criteria that require reporting using the Medwatch 3500A are:

- Any unexpected fatal or life threatening adverse experience associated with use of the drug must be reported to the FDA no later than 7 calendar days after initial receipt of the information [21 CFR 312.32(c)(2)]

- Any adverse experience associated with use of the drug that is both serious and unexpected must be submitted no later than 15 calendar days after initial receipt of the information [21 CFR 312.32(c)(1)]
- Any follow-up information to a study report shall be reported as soon as the relevant information becomes available. [21 CFR 312.32(d)(3)]

In addition, the study PI will submit annually within 60 days (via COH OIDRA) of the anniversary date of when the IND went into effect, an annual report to the FDA which is to include a narrative summary and analysis of the information of all FDA reports within the reporting interval, a summary report of adverse drug experiences, and history of actions taken since the last report because of adverse drug experiences.

7.3.3.2 Reporting to Osel, Inc

All serious adverse events and AESIs (initial and follow-up information) will be reported by the study PI to Osel, Inc within 24 hours.

8.0 AGENT INFORMATION

8.1 Nivolumab (NSC#748726); Opdivo

Product description:

Nivolumab will be supplied by City of Hope Comprehensive Cancer Center and billed to patients and/or their third-party payer.

Nivolumab injection is a clear opalescent, colorless to pale yellow, sterile, non-pyrogenic, single-use, isotonic aqueous solution formulated in sodium citrate, sodium chloride, mannitol, diethylenetriamine pentacetic acid (pentetic acid) and polysorbate 80 (Tween® 80), pH 6.0

Each vial is 100 mg (10 mg/mL) with a 0.7 mL overfill in 10 mL type I flint glass vials, with butyl rubber stoppers and aluminum seals

Solution preparation

Nivolumab can be infused undiluted (10 mg/mL) or diluted with 0.9% Sodium Chloride injection, USP or 5% dextrose, USP to drug concentrations no less than 0.35 mg/mL. Note: mix gently and do not shake. DO NOT administer as IV push or bolus injection.

Nivolumab injection is to be administered as a 30 minute IV infusion through a 0.2 micron to 1.2 micron pore size, low protein binding polyethersulfone membrane in-line filter.

Storage requirements

Vials of nivolumab injection must be stored at 2○C-8○C (36○F-46○F) and protected from light, freezing and shaking. If a storage temperature excursion is identified, promptly return Nivolumab to 2°C to 8°C and quarantine the supplies.

Stability:

No compatibilities between nivolumab and polyvinyl chloride (PVC), nonPVC DHEP (di(2-ethylhexyl)phthalate) IV components, or glass bottles have been observed.

The administration of undiluted and diluted solutions of Nivolumab must be completed within 24 hours preparation. If not used immediately, the infusion solution may be stored up to 24 hours in a refrigerator at 2○C-8○C (36○F-46○F) and a maximum of 4 hours of the total 24 hours can be at room temperature (20○C-25○C (68○F-77○F)) and room light. The maximum 4-hour period under room temperature and room light conditions includes the product administration period.

Route of administration:

Nivolumab injection is to be administered as a 30 minute IV infusion through a 0.2 micron to 1.2 micron pore size, low protein binding polyethersulfone membrane in-line filter.

The single-use dosage form contains no antibacterial preservative or bacteriostatic agent. Therefore, it is advised that the product be discarded 8 hours after initial entry.

No prophylactic medication should be given unless indicated by previous indication.

Availability:

Nivolumab is commercial agent supplied by City of Hope Comprehensive Cancer Center and billed to patients and/or their third-party payer.

8.2 Ipilimumab (NSC #732442); Yervoy

Product description:

Ipilimumab will be supplied by City of Hope Comprehensive Cancer Center and billed to patients and/or their third-party payer.

Ipilimumab injection is supplied as 200 mg/40 mL (5 mg/mL). It is formulated as a clear to slightly opalescent, colorless to pale yellow, sterile, non-pyrogenic, single-use, isotonic aqueous solution that may contain particles.

Each vial is a type I flint glass vial with gray butyl stoppers and sealed with aluminum seals. Ingredients are stated below.

Solution preparation

Ipilimumab is given undiluted (10 mg/mL) or further diluted in 0.9% Sodium Chloride injection, USP or 5% dextrose, USP in concentrations between 1 mg/mL and 4 mg/mL. Ipilimumab is stable in polyvinyl chloride (PVC), nonPVC DHEP (di(2-ethylhexyl)phthalate) IV bag or glass container up to 24 hours refrigerate at 2○C-8○C (36○F-46○F) or at room temperature/room light.

The product may be infused using a volumetric pump at the protocol specific dose(s), nonpyrogenic, low-protein-binding filter (pore size of 0.2 micrometer or 1.2 micrometer). DO NOT administer as IV push or bolus injection.

Storage requirements:

Vials of ipilimumab injection must be stored at 2○C-8○C (36○F-46○F) and protected from light, freezing. If a storage temperature excursion is identified, promptly return ipilimumab to 2○ to 8○C and quarantine the supplies.

Stability:

Vials of ipilimumab injection must be stored at 2○C-8○C (36○F-46○F) and protected from light, freezing.

Prepared IV ipilimumab solution is stable up to 24 hours refrigerated at 2○C-8○C (36○F-46○F) or at room temperature/room light.

Partially used vials or empty vials of ipilimumab injection should be discarded at the site according to appropriate drug disposal procedures.

Route of administration:

Ipilimumab should be administered as a short intravenous infusion over 30 minutes. No prophylactic medication should be given unless indicated by previous indication.

Availability:

Ipilimumab is commercial agent supplied by City of Hope Comprehensive Cancer Center and billed to patients and/or their third-party payer.

8.3 CBM588

Product description:

CBM588 will be supplied by Osel, Inc via the City of Hope Comprehensive Cancer Center Investigational Drug Pharmacy free of charge.

CBM588 fine granules are manufactured under cGMP at Miyarisan Pharmaceutical Company, Ltd. in Nagano, Japan. The product contains the bacterium *Clostridium butyricum* MIYAIRI 588, lactose, calcium carbonate, and corn starch. The fine granule preparation is light white-gray in color and has a characteristic odor and sweet taste. Each gram of fine granules contains 40 mg of CBM588 Powder, the active pharmaceutical ingredient, and 2×10^8 CFU of *C. butyricum*. CBM588 is packaged in cellophane polyethylene sachets containing 1 g of fine granules per sachet.

Storage requirements:

It is recommended that CBM588 be stored at controlled room temperature: the temperature maintained thermostatically that encompasses at the usual and customary working environment of 20°-25° (68°-77 °F). Excursions between 15° and 30° (59° and 86 °F) that are experienced in pharmacies, hospitals, and warehouses, and during shipping are allowed.

Stability:

CBM588 fine granules are stable for at least 18 months at 25 ± 2° C (77 ± 4° F) and 60 ± 5 % relative humidity and tested for appearance, particle size, identification, purity, loss on drying, and potency. The stability of all clinical product lots will be monitored for the duration of the clinical trial.

Route of administration:

CBM588 should be administered orally 2 times per day. Two sachets of CBM588 fine granules (2g) will be mixed with 100 ml of water and administered orally twice daily (morning and evening) for the duration of the study. The granules immediately dissolve in water, but form a cloudy solution due to the presence of precipitated calcium carbonate in the formulation.

Availability:

CBM588 is a commercial product in Japan manufactured by Miyarisan Pharmaceutical Co. Ltd. It is supplied as an investigational drug in the United States by Osel, Inc through the Investigational Drug Pharmacy at City of Hope Comprehensive Cancer Center.

9.0 CORRELATIVE/ SPECIAL STUDIES

9.1 Assessment of the Stool Microbiome

Samples will be collected at pre-specified time points as outlined in the study calendar, and specific instructions for stool collection will be supplied to the patient as per **Appendix B**. The following delineates methods of assessment.

9.1.1 Stool Collection

Fecal material will be collected using the OMNIgene Gut collection kit by patients at two time points, before starting treatment (baseline) and at the start of week 13. A standard operating procedure (SOP) has been generated for stool collection, as outlined in Appendix B. Stool collection kit contents are listed in Appendix B. A copy of this SOP will be provided to the patient and their understanding of the SOP will be documented by the PI.

All samples will be collected by participants at home and dropped to a FedEx location on the day of sample collection and prior to that day's final delivery. The sample will arrive at Dr. Sarah Highlander's laboratory at TGen North, to the attention of John Gillece (3051 W. Shamrell Blvd, Suite 106, Flagstaff, AZ, 86005) the next day.

9.1.2 Laboratory Processing and Analysis

Bacterial microbiome analysis

Stool mailed to City of Hope/TGen North will be processed and analyzed in the laboratory of Dr. Sarah Highlander. Total genomic DNA will be isolated from 0.25 g of feces using the PowerSoil DNA isolation kit (Mo Bio, USA). Purified DNA will be separated on a 1% agarose gel and quantified by densitometry and spectrophotometry (NanoDrop 1000; Thermo Scientific, USA). As described by Stearns et al, a PCR protocol will be used to amplify bacterial 16S rRNA genes from all samples.12 Following PCR primers including Illumina part of adapter sequences will be used to amplify V4 and V5 regions.

V4-F: ACACTCTTTCCCTACACGACGCTCTTCCGATCTGTGCCAGCMGCCGCGGTAA.

V4-R: ACACTCTTTCCCTACACGACGCTCTTCCGATCTGTGCCAGCMGCCGCGGTAA.

V5-F: ACACTCTTTCCCTACACGACGCTCTTCCGATCTGATTAGATACCCTGGTAG.

V5-R: GTGACTGGAGTTCAGACGTGTGCTCTTCCGATCTCCGTCAATTCMTTTGAGTTT.

Complete Illumina adapter and barcodes will be added by another 5 cycles of PCR to make Illumina library. After bioanalyzer and qPCR checking for QC. Multiple libraries will be mixed equally. Paired-end of sequencing (250bp or 300bp) will be performed by Illumina MiSeq V2/V3. Sequences will be clustered at a variety of percent identities using USEARCHalgorithm against the closed=reference.13 Taxonomy will then be assigned using the RDP 2.4 classifier as described in Smith et .al.

Illumina MiSeq high throughput sequencing will be used to sequence the 16S rRNA gene. Libraries will be constructed for all samples by amplification of V4 or V5 region of bacterial 16S rRNA. Barcodes will be created to uniquely index and label each sample for multiplex Illumina sequencing with paired end reads. Multiple test runs will be created first to ensure the validity in 16S rRNA amplification from the fecal samples and standard QC will be performed to examine the quality of multiplex Illumina sequencing. Low-quality reads will be removed and only reads that are perfectly match the assembly will be kept for further downstream analysis.

Fungal microbiome analysis

DNA will be extracted from stool samples using the MagMax PowerMicrobiome extraction kit using the KingFisher Flex magnetic purification system (ThermoFisher, Waltham, MA). All DNAs will be validated for purity and integrity by agarose gel electrophoresis, and fungal load will be quantitated using the FungiQuant TaqMan assay before proceeding to sequencing. DNAs will be subjected to internally transcribed spacer gene sequencing using the)ITS4-Fun AGCCTCCGCTTATTGATATGCTTAART and 5.8S-Fun AACTTTYRRCAAYGGATCWCT primers with dual-indexed bar codes as described by Kozich et al.[16-18] A DNA mock community that contains two fungal species will be sequenced as a positive control (Zymo, D6306) as will an extraction blank as a negative control. Libraries will be quantitated using KAPA Library Quantification Kit (KAPA Biosystems), normalized and pooled then sequenced on the Illumina MiSeq instrument using the v2 kit (2 x250 bp) targeting 20,000 reads per sample.

Demultiplexed ITS reads will be processed and clustered into operational taxonomic units (OTUs) using QIIME2.[19] Demultiplexed reads will be denoised using dada2 then clustered into OTUs using q2-dbotu. Taxonomic classification will be performed using feature-classifier classify-sklearn. Various QIIME2 plugins will be used to build a phylogenetic tree (phylogeny), calculate alpha diversity within samples (diversity plugin) and beta diversity between samples (diversity plugin). We have found that simple alpha diversity calculations, such as inverse Simpson diversity, Shannon and Chao estimates, and examination of the dominant genera in each sample provides a good first impression of the composition of the sample. We will use several different types of distance methods (Bray Curtis, UniFrac) to examine and compare the community distribution of the samples. These data will be examined by multidimensional scaling or principal components analysis and heatmaps will be created using hclust2.

Whole metagenome sequencing provides a complete picture of the taxonomic composition of a microbiome than does 16S rRNA gene or ITS sequencing and also permits predictions of microbiome function, which we can tie to metabolic and proteomic results. Metagenomics also has been used for the discovery of clinical biomarkers and will allow us to identify antibiotic resistance genes. Metagenomic DNA will be sequenced on the Illumina NextSeq platform to a depth of 2 Gb/sample.[20-21] Human reads will be identified by mapping them to the human genome GRCh38.p7 using BowTie2 and they will be removed. Demultiplexed reads will be quality trimmed using Trimmomatic to remove adapters and low-quality bases and reads.[22-23] Trimmed metagenomic reads will be taxonomically profiled using MetaPhlAn 2.0. Functional profiling of the metagenomes will be performed using HUMAnN2, which annotates open reading frames (ORFs) and generates gene family abundances, metabolic pathway coverage and abundances. Antimicrobial resistance genes will be identified by mapping to a curated version of the Resfams database. Resistance genes of interest will be verified by targeted amplicon sequencing.[24-27]

9.2 Assessment of Serum Cytokines

9.2.1 Research Blood Specimen Collection and Transfer to Processing Laboratory

One 10 mL CPT tube will be collected within 7 days before start of nivolumab and ipilimumab, during weeks 7, 13, 17 and 25 (+/- 1 week). The pre-treatment sample may be collected on the morning of initiation of therapy, so long as the sample precedes ipilimumab / nivolumab administration. Efforts will be made to collect the sample at the time of routine blood sample collection. Blood will be collected into 10 mL CPT vacuum tube, inverted slowly about 8-10 times, maintained at room temperature.

The sample will be labeled with the date, time of collection, IRB # ADD, and the participant's unique research participant number, prior to the prompt transport to the laboratory of Dr. Marcin Kortylewski for correlative analyses.

The Kortylewski laboratory should be notified of planned samples via email (mkortylewski@coh.org) or other laboratory designee preferably at least a day in advance of sample collection.

9.2.2 Initial Sample Processing – Isolation of PBMCs

Processing of samples will occur at in the laboratory of Dr. Marcin Kortylewski. The 10 mL CPT tube samples will be processed ASAP, ideally within a window of 4-6 hours. CPT tubes will be centrifuged at 1800 x g (approximately 2800 rpm on a Sorvall RT6000 centrifuge) for 20 minutes at room temperature. After centrifugation, plasma in the CPT tubes will be gently pipetted against the gel plug to dislodge cells stuck to the top of the gel. The cell suspension will be transferred to a 50 mL conical polypropylene tube. cRPMI will be added to a total of 40 mL. A 10 mL aliquot of cell suspension for counting will be removed. The 50 mL tubes will then be centrifuged at 250 x g for seven minutes at room temperature. When centrifugation is complete, the supernatant will be aspirated. PMBCs will be either cryopreserved or used fresh.

9.2.3 Sample Analysis

Analysis of samples for T-cell subpopulations will occur in the laboratory of Dr. Marcin Kortylewski. Relevant WBC subsets will be conducted through previously reported techniques. PBMCs will be immersed in a mixture of PBS, 2% FCS and 0.1% (wt/vol) sodium azide with Fc III/IIR-specific antibody to block nonspecific binding and stained the cells with different combinations of fluorochrome-coupled antibodies to CD11c, I-Ab (MHC class II), CD86, CD11b, Gr1, CD49b, CD3, CD25 or Lag-3, or with annexin V (BD Biosciences). We collected fluorescence data on FACSCalibur (Beckton Dickinson) and analyzed them using FlowJo software (Tree Star). This method has been previously published by Chalmin et al. [13]

10.0 STUDY CALENDAR

	Screening	Pre-tx	Nivolumab/Ipilimumab/CBM588				Nivolumab/CBM588			
			Wk 1	Wk 4	Wk 7	Wk 10	Wk 13	Wk 17	Wk 21	Wk 25+*
Informed consent	X									
Eligibility review	X									
Nivolumab			X	X	X	X	X	X	X	X[b]
Ipilimumab			X	X	X	X				
CBM588			X---X[b]							
Registration	X									
Participant orientation		X								
Research blood collection		X			X		X	X		X
Planned stool specimens		X					X			
Study log review/collection			X	X	X	X	X	X	X	X[b]
Data collection					X		X	X		X[b]
CT Chest/Abdomen/Pelvis	X[a]						X			X[b]
Bone scan	X[c]						X			X[b]
MRI or CT of Brain	X[d]									
Physical examination	X[e]		X	X	X	X	X	X	X	X
Safety Laboratory Assessments	X[f]		X	X	X	X	X	X	X	X

[a]Screening imaging should be completed within 6 weeks of registration. [b]Treatment administration, data collection, and response assessment should continue on 12-weekly intervals unless the treatment regimen is discontinued. [c]If indicated by the presence of bony metastasis at baseline. [d]Brain imaging should be performed if patients are symptomatic or have a prior history of CNS metastases. See Section 3.2.1 for criteria related to enrollment for patients with brain metastases. [e]Routine physical examination during clinic visit with the oncologist or nurse practitioner. [f]Screening phase safety labs are per standard of care (i.e. the following labs are due at baseline: CBC+diff, CMP, LDH, TSH, FT4, cortisol); treatment phase safety labs are per standard of care (i.e. the following labs are due on D1 of each cycle: CBC+diff, CMP, LDH, TSH, FT4, cortisol)

11.0 ENDPOINT DEFINITIONS/MEASUREMENT OF EFFECT

11.1 Change in *Bifidobacterium* composition of stool from baseline to week 13 of therapy

Methods of assessment for stool microbiome composition are noted in Section 9.1. We will assess the proportion *Bifidobacterium spp* at baseline (relative to the cumulative assessment of microbial species) and compare this to the proportion observed after completion of 12 weeks of therapy.

11.2 Best overall response, by RECIST criteria

Response is a secondary endpoint in this trial. For this purpose of this study, patients should be re-evaluated for response every 12 weeks. Response and progression will be evaluated in this study using the new international criteria proposed by the revised Response Evaluation Criteria in Solid Tumors (RECIST) guideline (version 1.1).[14] The published RECIST document is available at http://www.eortc.be/RECIST. Changes in the largest diameter (unidimensional measurement) of the tumor lesions and the shortest diameter in the case of malignant lymph nodes are used in the RECIST criteria.

11.2.1 Definitions

Evaluable for toxicity. All patients will be evaluable for toxicity from the time of their first treatment with VV2003 alone or in combination with and nivolumab and ipilumumab

Evaluable for objective response. Only those patients who have measurable disease present at baseline, have received at least one cycle of therapy, and have had their disease re-evaluated will be considered evaluable for response. These patients will have their response classified according to the definitions stated below. (Note: Patients who exhibit objective disease progression prior to the end of cycle 1 will also be considered evaluable.)

Evaluable Non-Target Disease Response. Patients who have lesions present at baseline that are evaluable but do not meet the definitions of measurable disease, have received at least one cycle of therapy, and have had their disease re-evaluated will be considered evaluable for non-target disease. The response assessment is based on the presence, absence, or unequivocal progression of the lesions.

11.2.2 Disease Parameters

Measurable disease. Measurable lesions are defined as those that can be accurately measured in at least one dimension (longest diameter to be recorded) as ≥20 mm by chest x-ray or as ≥10 mm with CT scan, MRI, or calipers by clinical exam. All tumor measurements must be recorded in millimeters (or decimal fractions of centimeters).

Tumor lesions that are situated in a previously irradiated area are considered measurable.

Malignant lymph nodes. To be considered pathologically enlarged and measurable, a lymph node must be ≥15 mm in short axis when assessed by CT scan (CT scan slice thickness recommended to be no greater than 5 mm). At baseline and in follow-up, only the short axis will be measured and followed.

Non-measurable disease. All other lesions (or sites of disease), including small lesions (longest diameter <10 mm or pathological lymph nodes with ≥10 to <15 mm short axis), are considered non-measurable disease. Bone lesions, leptomeningeal disease, ascites, pleural/pericardial

effusions, lymphangitis cutis/pulmonitis, inflammatory breast disease, and abdominal masses (not followed by CT or MRI), are considered as non-measurable.

Target lesions. All measurable lesions up to a maximum of 2 lesions per organ and 5 lesions in total, representative of all involved organs, should be identified as target lesions and recorded and measured at baseline. Target lesions should be selected on the basis of their size (lesions with the longest diameter), be representative of all involved organs, but in addition should be those that lend themselves to reproducible repeated measurements. It may be the case that, on occasion, the largest lesion does not lend itself to reproducible measurement in which circumstance the next largest lesion, which can be measured reproducibly should be selected. A sum of the diameters (longest for non-nodal lesions, short axis for nodal lesions) for all target lesions will be calculated and reported as the baseline sum diameters. If lymph nodes are to be included in the sum, then only the short axis is added into the sum. The baseline sum diameters will be used as reference to further characterize any objective tumor regression in the measurable dimension of the disease.

Non-target lesions. All other lesions (or sites of disease) including any measurable lesions over and above the 5 target lesions should be identified as non-target lesions and should also be recorded at baseline. Measurements of these lesions are not required, but the presence, absence, or in rare cases unequivocal progression of each should be noted throughout follow-up.

11.2.3 Methods for Evaluation of Measurable Disease

All measurements should be taken and recorded in metric notation using a ruler or calipers. All baseline evaluations should be performed as closely as possible to the beginning of treatment and never more than 4 weeks before the beginning of the treatment.

The same method of assessment and the same technique should be used to characterize each identified and reported lesion at baseline and during follow-up. Imaging-based evaluation is preferred to evaluation by clinical examination unless the lesion(s) being followed cannot be imaged but are assessable by clinical exam.

Clinical lesions: Clinical lesions will only be considered measurable when they are superficial (e.g., skin nodules and palpable lymph nodes) and ≥10 mm diameter as assessed using calipers (e.g., skin nodules). In the case of skin lesions, documentation by color photography, including a ruler to estimate the size of the lesion, is recommended.

Chest x-ray: Lesions on chest x-ray are acceptable as measurable lesions when they are clearly defined and surrounded by aerated lung. However, CT is preferable.

Conventional CT and MRI: This guideline has defined measurability of lesions on CT scan based on the assumption that CT slice thickness is 5 mm or less. If CT scans have slice thickness greater than 5 mm, the minimum size for a measurable lesion should be twice the slice thickness. MRI is also acceptable in certain situations (e.g. for body scans).

Use of MRI remains a complex issue. MRI has excellent contrast, spatial, and temporal resolution; however, there are many image acquisition variables involved in MRI, which greatly impact image quality, lesion conspicuity, and measurement. Furthermore, the availability of MRI is variable globally. As with CT, if an MRI is performed, the technical specifications of the scanning sequences used should be optimized for the evaluation of the type and site of disease. Furthermore, as with CT, the modality used at follow-up should be the same as was used at baseline and the lesions should be measured/assessed on the same pulse sequence. It is beyond the scope of the RECIST

guidelines to prescribe specific MRI pulse sequence parameters for all scanners, body parts, and diseases. Ideally, the same type of scanner should be used and the image acquisition protocol should be followed as closely as possible to prior scans. Body scans should be performed with breath-hold scanning techniques, if possible.

PET-CT: At present, the low dose or attenuation correction CT portion of a combined PET-CT is not always of optimal diagnostic CT quality for use with RECIST measurements. However, if the site can document that the CT performed as part of a PET-CT is of identical diagnostic quality to a diagnostic CT (with IV and oral contrast), then the CT portion of the PET-CT can be used for RECIST measurements and can be used interchangeably with conventional CT in accurately measuring cancer lesions over time. Note, however, that the PET portion of the CT introduces additional data which may bias an investigator if it is not routinely or serially performed.

Ultrasound: Ultrasound is not useful in assessment of lesion size and should not be used as a method of measurement. Ultrasound examinations cannot be reproduced in their entirety for independent review at a later date and, because they are operator dependent, it cannot be guaranteed that the same technique and measurements will be taken from one assessment to the next. If new lesions are identified by ultrasound in the course of the study, confirmation by CT or MRI is advised. If there is concern about radiation exposure at CT, MRI may be used instead of CT in selected instances.

11.2.4 Response Criteria

11.2.4.1 Evaluation of Target Lesions

Complete Response (CR): Disappearance of all target lesions. Any pathological lymph nodes (whether target or non-target) must have reduction in short axis to <10 mm.

Partial Response (PR): At least a 30% decrease in the sum of the diameters of target lesions, taking as reference the baseline sum diameters.

Progressive Disease (PD): At least a 20% increase in the sum of the diameters of target lesions, taking as reference the smallest sum on study (this includes the baseline sum if that is the smallest on study). In addition to the relative increase of 20%, the sum must also demonstrate an absolute increase of at least 5 mm. (Note: the appearance of one or more new lesions is also considered progressions).

Stable Disease (SD): Neither sufficient shrinkage to qualify for PR nor sufficient increase to qualify for PD, taking as reference the smallest sum diameters while on study.

11.2.4.2 Evaluation of Non-Target Lesions

Complete Response (CR): Disappearance of all non-target lesions. All lymph nodes must be non-pathological in size (<10 mm short axis).

Non-CR/Non-PD: Persistence of one or more non-target lesion(s) and/or maintenance of tumor marker level above the normal limits.

Progressive Disease (PD): Appearance of one or more new lesions and/or unequivocal progression of existing non-target lesions. Unequivocal progression should not normally trump target lesion status. It must be representative of overall disease status change, not a single lesion increase.

Although a clear progression of "non-target" lesions only is exceptional, the opinion of the treating physician should prevail in such circumstances, and the progression status should be confirmed at a later time by the review panel (or Principal Investigator).

11.2.4.3 Evaluation of Best Overall Response

The best overall response is the best response recorded from the start of the treatment until disease progression/recurrence (taking as reference for progressive disease the smallest measurements recorded since the treatment started). The patient's best response assignment will depend on the achievement of both measurement and confirmation criteria.

11.3 Duration of time from enrollment to progression

PFS is defined as the duration of time from start of treatment to time of progression or death, whichever occurs first.

11.4 Comparison of the Shannon index (a measure of microbial diversity) from baseline to week 13 of therapy

Using translational methods described in Section 9.1, we will compute the Shannon index at baseline and at week 12 for a comparison of microbial diversity at these two timepoints.

11.5 Comparison of the proportion of circulating Tregs at baseline to levels of circulating Tregs on treatment

Using translational methods described in Section 9.2, we will estimate the proportion of Tregs in the blood. This will be assessed graphically across serial timepoints of blood collection (see Study Calendar) to ascertain any trends.

11.6 Comparison of the proportion of circulating MDSCs at baseline to levels of circulating MDSCs on treatment

Using translational methods described in Section 9.2, we will estimate the proportion of MDSCs in the blood. This will be assessed graphically across serial timepoints of blood collection (see Study Calendar) to ascertain any trends.

11.7 Comparison of IL-6, IL-8 and other cytokines at baseline to levels of the same cytokines on treatment

Using translational methods described in Section 9.2, we will estimate the proportion of serum cytokines in the blood. This will be assessed graphically across serial timepoints of blood collection (see Study Calendar) to ascertain any trends.

12.0 STATISTICAL CONSIDERATIONS

12.1 Study Design

This is a randomized study of nivolumab/ipilimumab alone or in combination with CBM588. The objective is to define the biologic effect of CBM588 when used in combination with nivolumab/ipilimumab. Our preclinical data suggests that *Bifidobacterium spp* are associated with responses to immunotherapy, and

we hypothesize that CBM588 will increase levels of *Bifidobacterium spp*. We will compare the proportional increase in *Bifidobacterium spp* with the addition of CBM588 to CBM588 and identify the cohort that achieves the largest such increase relative to patients receiving nivolumab/ipilimumab alone.

12.2 **Sample Size and Accrual Rate**

We will randomize 30 patients in a 1:2 fashion to receive nivolumab/ipilimumab alone [Arm 1] or with CBM588 [Arm 2]. We anticipate accrual of 30 patients over a 2 year span (approximately 1.5 patients per month), with approximately 12 months of follow-up on average (based on PFS estimates for nivolumab/ipilimumab). Given an anticipated 80% rate of consent to this study based on existing studies in patients with newly diagnosed mRCC, we would have to approach approximately 2 patients per month. This is feasible with current rates of new patient volume at our institution.

12.3 **Statistical Analysis Plan**

Primary Endpoint

- (1) Change in Bifidobacterium composition of stool from baseline to week 12 of therapy Comparison of the Shannon index (a measure of microbial diversity) from baseline to week 12 of therapy
- Analysis plan: Change in the *Bifidobacterium* from baseline to week 12 will be assessed for patients on both arms. With 20 on the CBM588 containing arm, and 10 on the non-CBM588 containing arm, we will have 80% power to detect a 1 standard deviation (common standard deviation of the change in *Bifidobacterium)* difference between the mean change detected in the two groups using a two-group t-test with a one-sided type I error of 0.05.

Secondary Endpoints

- (1a) Comparison of the Shannon index (a measure of microbial diversity) from baseline to week 12 of therapy will be conducted in a similar fashion. As this is a secondary measure, any conclusions will discuss the multiple comparison issue inherent in this second analysis.

- (1b) Best overall response, by RECIST criteria, with nivolumab/ipilimumab alone versus nivolumab/ipilimumab with CBM588.

Analysis plan: The association between treatment arm and overall response as per RECIST criteria (response observed vs not observed) will be examined using Fisher's exact test

- (1c) Progression-free survival (PFS), assessed as the duration of time from enrollment to progression, with nivolumab/ipilimumab alone versus nivolumab/ipilimumab with CBM588

Analysis plan: The difference in progression free survival across the two groups will be explored graphically using Kaplan-Meier survival plots. Median progression-free survival time for each of the two arms will be reported and Cox Proportional Hazards model will be used to estimate the hazard ratio and its confidence interval.

We will also conduct an exploratory analysis of the following, with no adjustment for the multiple comparison issue although any conclusion will include a discussion of the limitations of any conclusions drawn due to the multiple comparisons concern:

- (2a) Comparison of the proportion of circulating Tregs at baseline to levels of circulating Tregs with nivolumab/ipilimumab alone versus nivolumab/ipilimumab with CBM588
- (2b) Comparison of the proportion of circulating MDSCs with nivolumab/ipilimumab alone versus nivolumab/ipilimumab with CBM588
- (2c) Comparison of IL-6, IL-8 and other cytokines/chemokines with nivolumab/ipilimumab alone versus nivolumab/ipilimumab with CBM588.
- (3) Comparison of toxicities such as diarrhea and nausea using CTCAE v5 criteria with nivolumab/ipilimumab alone versus nivolumab/ipilimumab with CBM588.

Safety Endpoints/Early Stopping:

- In Checkmate 214, 8/547 patients died due to treatment-related complications (~1%). As a result any treatment-related death on the combination arm with CBM588 will hold the study pending detailed review and an evaluation of the cause of the toxicity, with accrual allowed to continue pending approval of the COH DSMC. A second treatment-related death on the CBM588 arm closes the study to further accrual.
- Early stopping for adverse events in the context of the study is difficult due to 46% of patients on Checkmate 214 treated with ipilimumab and nivolumab experience grade 3 or 4 events, and the potential for the trade-off between toxicity and activity with CBM588 gut manipulation. However, if the number unable to receive all four doses of ipilimumab increases from 20% to 60%, the response rate will not likely exceed Checkmate 214. As a result, if after 10 patients have accrued to the CBM588 arm, if 5 or more patients are unable to receive all four doses of ipilimumab, the study will hold accrual pending review of both toxicity and clinical activity. The likelihood of a 20% rate of treatment discontinuation in the first 4 cycles resulting in 5 or more patients with treatment discontinuation in the first 10 patients is less than 5%.

13.0 PROTOCOL DEVIATIONS AND SINGLE SUBJECT EXCEPTIONS

It is understood that deviations from the protocol should be avoided, except when necessary to eliminate an immediate hazard to a research participant. Brief interruptions and delays may occasionally be required because of travel delays, airport closures, inclement weather, family responsibilities, security alerts, government holidays, and so forth. Delays can also extend to complications of disease or unrelated medical illnesses not related to disease progression. The PI has the discretion to deviate from the protocol when necessary so long as such a deviation does not threaten patient safety or protocol scientific integrity. As a result of deviations, corrective actions are to be developed by the study staff and implemented promptly

13.1 Definitions

13.1.1 Deviation

A deviation is a divergence from a specific element of a protocol that occurred without prior IRB approval. Investigators may deviate from the protocol to eliminate immediate hazard(s) for the protection, safety, and well-being of the study subjects without prior IRB approval. Examples include, but are not limited to: a) dose adjustments based on excessive patient weight; b) alteration in treatment schedule due to non-availability of the research participant for treatment; and c) laboratory test results which are slightly outside the protocol requirements but at levels that do not affect participant safety.

13.1.2 Single Subject Exceptions (SSE)

An SSE is a planned deviation, meaning that it involves circumstances in which the specific procedures called for in a protocol are not in the best interests of a specific patient. It is a deviation that is anticipated and receives prior approval by the Principal Investigator and the COH IRB.

13.2 Reporting of Deviations and SSEs

13.2.1 Reporting Deviations

For any deviation, the Investigator will notify the COH DSMC and IRB within 5 calendar days of its occurrence via iRIS in accordance with the Clinical Research Protocol Deviation policy.

13.2.2 Reporting Single Subject Exceptions

The SSE must be submitted as a “Single Subject Exception Amendment Request” via iRIS in accordance with IRB guidelines and the Clinical Research Protocol Deviation policy. An IRB approved SSE does not need to be submitted as a deviation to the DSMC.

In addition, if contractually obligated, the sponsor must also approve the deviation.

14.0 STUDY OVERSIGHT, QUALITY ASSURANCE, AND DATA & SAFETY MONITORING

14.1 All Investigator Responsibilities

An investigator is responsible for ensuring that an investigation is conducted according to the signed investigator statement, the investigational plan, and applicable regulations; for protecting the rights, safety, and welfare of subjects under the investigator's care; and for the control of drugs under investigation.

All Investigators agree to:

- Conduct the study in accordance with the protocol and only make changes after notifying the Sponsor (or designee), except when necessary to protect the safety, rights or welfare of subjects.
- Personally conduct or supervise the study (or investigation).
- Ensure that the requirements relating to obtaining informed consent and IRB review and approval meet federal guidelines, as stated in § 21 CFR, parts 50 and 56.
- Report to the Sponsor or designee any AEs that occur in the course of the study, in accordance with §21 CFR 312.64.
- Ensure that all associates, colleagues and employees assisting in the conduct of the study are informed about their obligations in meeting the above commitments.
- Maintain adequate and accurate records in accordance with §21 CFR 312.62 and to make those records available for inspection with the Sponsor (or designee).
- Ensure that an IRB that complies with the requirements of §21 CFR part 56 will be responsible for initial and continuing review and approval of the clinical study.
- Promptly report to the IRB and the Sponsor all changes in the research activity and all unanticipated problems involving risks to subjects or others (to include amendments and IND safety reports).
- Seek IRB and Sponsor approval before any changes are made in the research study, except when necessary to eliminate hazards to the patients/subjects.
- Comply with all other requirements regarding the obligations of clinical investigators and all other pertinent requirements listed in § 21 CFR part 312.

14.2 Study Principal Investigator Responsibilities

The Study Principal Investigator is responsible for the conduct of the clinical trial, including overseeing that sponsor responsibilities as defined in § 21 CFR 312. Subpart D are executed in accordance with federal regulations.

14.3 Protocol Management Team (PMT)

The Protocol Management Team (PMT), minimally consisting of the study PI, collaborating investigators, research nurse, clinical research associate/coordinator, and the study biostatistician, is responsible for ongoing monitoring of the data and safety of this study, including implementation of the stopping rules for safety/toxicity.

The PMT is recommended to meet (in person or via teleconference) at least monthly to review study status. This review will include, but not be limited to, reportable AEs and UPs, and an update of the ongoing study summary that describes study progress in terms of the study schema. The meeting will be a forum to discuss study related issues including accrual, SAE/AEs experienced, study response, deviations/violations and study management issues. The appropriateness of further subject enrollment and the specific intervention for subsequent subject enrollment are addressed. It is recommended that minutes of these discussions be taken to document the date of these meetings, attendees and the issues that were discussed (in a general format).

14.4 Monitoring

Clinical site monitoring is conducted to ensure that the rights of human subjects are protected, that the study is implemented in accordance with the protocol and regulatory requirements, and that the quality

and integrity of study data and data collection methods are maintained. Monitoring for this study will be performed by the City of Hope Office of Clinical Trials Auditing and Monitoring (OCTAM).

The Investigator will permit the study monitors and appropriate regulatory authorities direct access to the study data and to the corresponding source data and documents to verify the accuracy of this data. The Investigator will allocate adequate time for such monitoring activities. The Investigator will also ensure that the monitor or other compliance or quality assurance reviewer is given access to all the above noted study-related documents and study related facilities (e.g. pharmacy, diagnostic laboratory, etc.), and has adequate space to conduct the monitoring visit.

Details of clinical site monitoring are documented in the OCTAM SOP. This document specifies the frequency of monitoring, monitoring procedures, the level of clinical site monitoring activities (e.g., the percentage of subject data to be reviewed), and the distribution of monitoring reports. Staff from OCTAM will conduct monitoring activities and provide reports of the findings and associated action items in accordance with the details described in the SOP. Documentation of monitoring activities and findings will be provided to the study team, and the COH DSMC.

14.5 Quality Assurance

The City of Hope Clinical Research Information Support will provide support for this multi-center trial as detailed in the COH DCC Operations Plan provided as a supplement to this document.

14.6 City of Hope Data and Safety Monitoring Committee

This is a risk level 4 study as defined in the City of Hope Institutional Data and Safety Monitoring Plan. This determination was made because the study involves a COH held IND.

The DSMC is a multidisciplinary committee charged with overseeing the monitoring of safety of participants in clinical trials, and the conduct, progress, validity, and integrity of the data for all clinical trials that are sponsored by City of Hope. The committee is composed of clinical specialists with experience in oncology and who have no direct relationship with the study. The committee reviews the progress and safety of all active research protocols that are not monitored by another safety and data monitoring committee or board.

The Study Principal Investigator is required to submit periodic status reports (the PMT report) according to the guidelines outlined in the City of Hope Institutional Data and Safety Monitoring Plan. The PMT report will be submitted to the COH DSMC semi-annually from the date of activation.

The COH Data and Safety Monitoring Committee (DSMC) will review and monitor toxicity and accrual data from this trial. The DSMC will review up-to-date participant accrual; summary of all adverse events captured via routine and expedited reporting; a summary of deviations; any response information; monitoring reports, and summary comments provided by the study team. Other information (e.g. scans, laboratory values) will be provided upon request. For Phase I studies, a Phase I Tracking Log will be utilized and reviewed by the DSMC to monitor data and safety for dose escalation. A review of outcome results (response, toxicity and adverse events) and factors external to the study (such as scientific or therapeutic developments) is discussed, and the Committee votes on the status of each study. Information that raises any questions about participant safety will be addressed with the Principal Investigator, statistician and study team.

15.0 ETHICAL AND REGULATORY CONSIDERATIONS

15.1 Patient Protection

The responsible investigator will ensure that this study is conducted in agreement with either the Declaration of Helsinki (Tokyo, Venice, Hong Kong, Somerset West and Edinburgh amendments) or the laws and regulations of the country, whichever provides the greatest protection of the patient.
The protocol has been written, and the study will be conducted according to the principles of Guideline for Good Clinical Practice.

(ref:http://www.ich.org/fileadmin/Public_Web_Site/ICH_Products/Guidelines/Efficacy/E6_R1/Step4/E6_R1__Guideline.pdf)

The protocol will be approved by local, centralized, regional or national ethics committees / institutional review boards.

15.2 Subject Identification

This research will be conducted in compliance with federal and state of California requirements relating to protected health information (PHI).

All samples will be coded prior to submission to the research laboratories. The coded identifier will be the COH research patient number (RPN), provided by the MIDAS system, which is devoid of direct participant identifiers. The key to the code is maintained in MIDAS which is a secure environment.
All study related forms including consent documents and patient diaries will be stored in locked and secure locations.

Medical records of participants will be securely maintained in the strictest confidence, according to current legal requirements. All information will be treated confidentially. No identifiers will be used in any subsequent publication of these results.

15.3 Informed Consent

All participants will undergo standard written informed consent procedures as dictated by the City of Hope Human Research Protections Office prior to performing any screening procedures that are not part of standard-of-care. Informed consent will be obtained by the principal investigator, collaborating investigators, or other IRB designated personnel who will meet the training requirements established by the IRB.

In addition, they will review the experimental subject's bill of rights and the HIPAA research authorization form.

All patients will be informed of the aims of the study, the possible adverse events, the procedures and possible hazards to which he/she will be exposed, and the mechanism of treatment allocation. They will be informed as to the strict confidentiality of their patient data, but that their medical records may be

reviewed for trial purposes by authorized individuals other than their treating physician. An example of a patient informed consent statement is given as an appendix to this protocol.

Prospective research participants will be afforded sufficient time to consider whether or not to participate in the research.

It is the responsibility of the individual investigator to translate the enclosed informed consent document. The translated version should be dated and version controlled.

The bold sections of the enclosed informed consent document are the sections that must appear in the translation.

The translated informed consent form is part of the documents to be submitted to the ethics committee for approval. The competent ethics committee for each institution must validate local informed consent documents before the center can join the study. It is the responsibility of the Local Ethical Committee to guarantee that the translation is conforming to the ICH-GCP guidelines.

It will be emphasized that the participation is voluntary and that the patient is allowed to refuse further participation in the protocol whenever he/she wants. This will not prejudice the patient's subsequent care. Documented informed consent must be obtained for all patients included in the study before they are registered or randomized in the study. This must be done in accordance with the national and local regulatory requirements.

16.0 REFERENCES

1. Surveillance, Epidemiology, and End Results Program. SEER stat fact sheets: kidney and renal pelvis cancer. Bethesda, MD, National Cancer Institute

2. Choueiri TK, Motzer RJ: Systemic Therapy for Metastatic Renal-Cell Carcinoma. N Engl J Med 376:354-366, 2017

3. Motzer RJ, Tannir NM, McDermott DF, et al: Nivolumab plus Ipilimumab versus Sunitinib in Advanced Renal-Cell Carcinoma. N Engl J Med 378:1277-1290, 2018

4. Motzer RJ, Tannir NM, McDermott DF, et al: Nivolumab plus Ipilimumab versus Sunitinib in Advanced Renal-Cell Carcinoma. New England Journal of Medicine 378:1277-1290, 2018

5. Vetizou M, Pitt JM, Daillere R, et al: Anticancer immunotherapy by CTLA-4 blockade relies on the gut microbiota. Science 350:1079-84, 2015

6. Sivan A, Corrales L, Hubert N, et al: Commensal Bifidobacterium promotes antitumor immunity and facilitates anti-PD-L1 efficacy. Science 350:1084-9, 2015

7. Gopalakrishnan V, Spencer CN, Nezi L, et al: Gut microbiome modulates response to anti-PD-1 immunotherapy in melanoma patients. Science 359:97-103, 2018

8. Derosa L, Hellmann MD, Spaziano M, et al: Negative association of antibiotics on clinical activity of immune checkpoint inhibitors in patients with advanced renal cell and non-small-cell lung cancer. Ann Oncol 29:1437-1444, 2018

9. Isa K, Oka K, Beauchamp N, et al: Safety assessment of the Clostridium butyricum MIYAIRI 588(R) probiotic strain including evaluation of antimicrobial sensitivity and presence of Clostridium toxin genes in vitro and teratogenicity in vivo. Hum Exp Toxicol 35:818-32, 2016

10. Seki H, Shiohara M, Matsumura T, et al: Prevention of antibiotic-associated diarrhea in children by Clostridium butyricum MIYAIRI. Pediatr Int 45:86-90, 2003

11. Yasueda A, Mizushima T, Nezu R, et al: The effect of Clostridium butyricum MIYAIRI on the prevention of pouchitis and alteration of the microbiota profile in patients with ulcerative colitis. Surg Today 46:939-49, 2016

12. Sato S, Nagai H, Igarashi Y: Effect of probiotics on serum bile acids in patients with ulcerative colitis. Hepatogastroenterology 59:1804-8, 2012

13. Chalmin F, Ladoire S, Mignot G, et al: Membrane-associated Hsp72 from tumor-derived exosomes mediates STAT3-dependent immunosuppressive function of mouse and human myeloid-derived suppressor cells. J Clin Invest 120:457-71, 2010

14. Eisenhauer EA, Therasse P, Bogaerts J, et al: New response evaluation criteria in solid tumours: revised RECIST guideline (version 1.1). Eur J Cancer 45:228-47, 2009

15. Oken MM, Creech RH, Tormey DC, et al: Toxicity and response criteria of the Eastern Cooperative Oncology Group. Am J Clin Oncol 5:649-55, 1982

16. Liu CM, Kachur S, Dwan MG, Abraham AG, Aziz M, Hsueh PR, Huang YT, Busch JD, Lamit LJ, Gehring CA et al: FungiQuant: a broad-coverage fungal quantitative real-time PCR assay. BMC Microbiol 2012, 12:255.

17. Taylor DL, Walters WA, Lennon NJ, Bochicchio J, Krohn A, Caporaso JG, Pennanen T: Accurate Estimation of Fungal Diversity and Abundance through Improved Lineage-Specific Primers Optimized for Illumina Amplicon Sequencing. Appl Environ Microbiol 2016, 82(24):7217-7226.

18. Kozich JJ, Westcott SL, Baxter NT, Highlander SK, Schloss PD: Development of a dual-index sequencing strategy and curation pipeline for analyzing amplicon sequence data on the MiSeq Illumina sequencing platform. Appl Environ Microbiol 2013, 79(17):5112-5120.

19. Bolyen E, Rideout JR, Dillon MR, Bokulich NA, Abnet CC, Al-Ghalith GA, Alexander H, Alm EJ, Arumugam M, Asnicar F et al: Reproducible, interactive, scalable and extensible microbiome data science using QIIME 2. Nat Biotechnol 2019, 37(8):852-857.

20. Wen C, Zheng Z, Shao T, Liu L, Xie Z, Le Chatelier E, He Z, Zhong W, Fan Y, Zhang L et al: Quantitative metagenomics reveals unique gut microbiome biomarkers in ankylosing spondylitis. Genome Biol 2017, 18(1):142.

21. Yu J, Feng Q, Wong SH, Zhang D, Liang QY, Qin Y, Tang L, Zhao H, Stenvang J, Li Y et al: Metagenomic analysis of faecal microbiome as a tool towards targeted non-invasive biomarkers for colorectal cancer. Gut 2017, 66(1):70-78.

22. Langmead B, Salzberg SL: Fast gapped-read alignment with Bowtie 2. Nat Methods 2012, 9(4):357-359.

23. Bolger AM, Lohse M, Usadel B: Trimmomatic: a flexible trimmer for Illumina sequence data. Bioinformatics 2014, 30(15):2114-2120.

24. Segata N, Waldron L, Ballarini A, Narasimhan V, Jousson O, Huttenhower C: Metagenomic microbial community profiling using unique clade-specific marker genes. Nat Methods 2012, 9(8):811-814.

25. Truong DT, Franzosa EA, Tickle TL, Scholz M, Weingart G, Pasolli E, Tett A, Huttenhower C, Segata N: MetaPhlAn2 for enhanced metagenomic taxonomic profiling. Nat Methods 2015, 12(10):902-903.

26. Franzosa EA, McIver LJ, Rahnavard G, Thompson LR, Schirmer M, Weingart G, Lipson KS, Knight R, Caporaso JG, Segata N et al: Species-level functional profiling of metagenomes and metatranscriptomes. Nat Methods 2018, 15(11):962-968.

27. Gibson MK, Forsberg KJ, Dantas G: Improved annotation of antibiotic resistance determinants reveals microbial resistomes cluster by ecology. ISME J 2015, 9(1):207-216.

APPENDIX A: PERFORMANCE STATUS CONVERSION CHART

ECOG PERFORMANCE STATUS	KARNOFSKY PERFORMANCE STATUS
0—Fully active, able to carry on all pre-disease performance without restriction	100—Normal, no complaints; no evidence of disease<br><br>90—Able to carry on normal activity; minor signs or symptoms of disease
1—Restricted in physically strenuous activity but ambulatory and able to carry out work of a light or sedentary nature, e.g., light house work, office work	80—Normal activity with effort, some signs or symptoms of disease<br><br>70—Cares for self but unable to carry on normal activity or to do active work
2—Ambulatory and capable of all selfcare but unable to carry out any work activities; up and about more than 50% of waking hours	60—Requires occasional assistance but is able to care for most of personal needs<br><br>50—Requires considerable assistance and frequent medical care
3—Capable of only limited selfcare; confined to bed or chair more than 50% of waking hours	40—Disabled; requires special care and assistance<br><br>30—Severely disabled; hospitalization is indicated although death not imminent
4—Completely disabled; cannot carry on any selfcare; totally confined to bed or chair	20—Very ill; hospitalization and active supportive care necessary<br><br>10—Moribund
5—Dead	0—Dead

APPENDIX B: STOOL COLLECTION PROCEDURE

STOOL COLLECTION KIT GENERAL INSTRUCTIONS

As a part of your participation in the current study, we have some specific instructions related to collection of stool. Please abide by these instructions, as they are essential for the proper conduct of the study.

TOOL SAMPLING USING THE OMNIgene GUT Kit

Read all instructions prior to sample collection.

Samples should ONLY be collected Sunday-Wednesday and shipped Monday-Wednesday.

If you have diarrhea, wait until the next bowel movement to collect the sample.

Your collection kit will be composed of the following:

☐	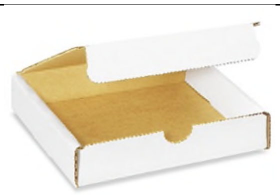	Shipment box – save to return the sample
☐	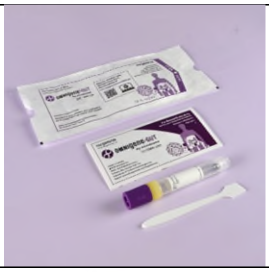	OMNIgene•GUT Kit Collection Kit
☐	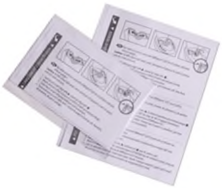	Toilet Accessory
☐	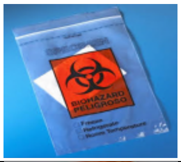	Plastic Biohazard Specimen Bag with Absorbent Paper
☐	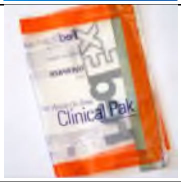	Plastic FedEx Clinical Pak
☐	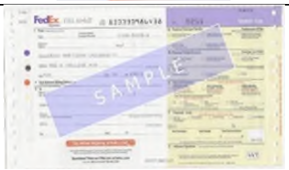	Preprinted FedEx Shipping Label

If any part of the kit is missing contact Paulo Bergerot at 626-218-3504 immediately to obtain the missing materials.

Stool Collection Instructions:

1. Read the instructions provided in the OMNIgene�GUT Kit Collection Kit before beginning stool collection. You can watch the instructional video on YouTube.
2. Wash with water if the stabilizing liquid comes in contact with skin or eyes.
3. Wash your hands thoroughly.
4. Empty your bladder prior to collection.
5. Use the Toilet Accessory (Appendix A) to collect stool free from toilet water and urine. Follow the instructions provided with the Toilet Accessory. DO NOT use toilet paper for collection.
6. Collect your stool sample from the Toilet Accessory following the OMNIgene�GUT instructions (Appendix B) included with the kit. DO NOT push the stool into the tube. Only a small amount of sample is needed. Be sure that the purple cap is screwed on tightly.
7. Shake the tube vigorously for at least 30 seconds or until all of the solids have been dispersed into suspension
8. Place the tube in the Biohazard Specimen Bag and seal. Write the date and time of collection on the label on the bag.
9. Wash your hands thoroughly.
10. Place the Biohazard Specimen Bag in the Shipping Box.
11. Place the box inside the FedEx Clinical Pak. Affix the preprinted FedEx shipping label to the outside of the Clinical Pak.
12. Follow the Shipping Instructions on the following page. Return the sample within 24 hours of collection. Only ship Monday-Wednesday.

FedEx Shipment of Biological Samples

1. You may drop off your Clinical Pak at a staffed FedEx location (NOT a drop box). Remember that samples should remain at ambient temperature.

THANK YOU!

If you have any questions about any of these procedures do not hesitate to contact the study coordinator at (626) 218-3504 or microbiome@tgen.org.

OM-AC1 Toilet accessory

Used with OMNIgene•GUT

USER INSTRUCTIONS

This toilet accessory is used with OMNIgene®•GUT kit to facilitate collection of fecal samples.

Caution: Do NOT allow toilet water, urine, detergent or fragrance to come in contact with this toilet accessory.

Storage: 15°C to 25°C

Procedure:

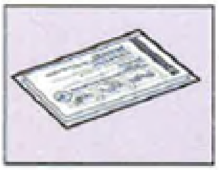

1 Two toilet accessories are provided in the event that you are unable to collect a sample on the first attempt.

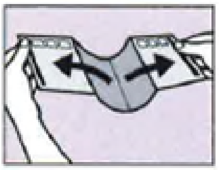

2 Carefully peel open edge with ▲; repeat for edge with ■.

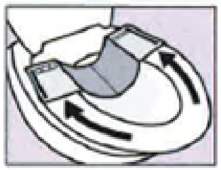

3 Attach adhesive surface of accessory to **BACK of toilet seat** with adhesive close to the outer edge of the seat and press firmly.

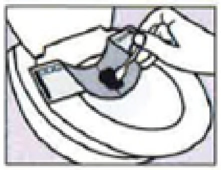

4 Collect fecal sample following OMNIgene•GUT user instructions.

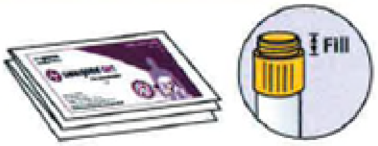

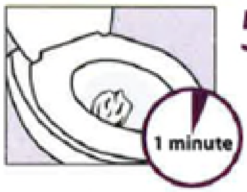

5 Drop used accessory into toilet. Wait 1 minute for paper to become soft, then flush. Alternatively, discard in garbage.

DNA genotek

Superior samples
Proven performance

OM-AC1 is made in Netherlands for DNA Genotek Inc.
3000 - 500 Palladium Drive
Ottawa, ON, Canada K2V 1C2

Toll-free (North America): 1.866.813.6354
Tel.: +1.613.723.5757 • Fax: +1.613.723.5057
info@dnagenotek.com
www.dnagenotek.com

*OMNIgene is a registered trademark of DNA Genotek Inc.
Some DNA Genotek products may not be available in all geographic regions, contact your sales representative for details.
All DNA Genotek protocols, white papers and application notes, are available in the support section of our website at www.dnagenotek.com.

Patent (www.dnagenotek.com/legalnotices)
© 2017 DNA Genotek Inc., a subsidiary of OraSure Technologies, Inc., all rights reserved.
PD-PR-00684 Issue 1/2017-05

CE

OMNIgene•GUT

For microbiome

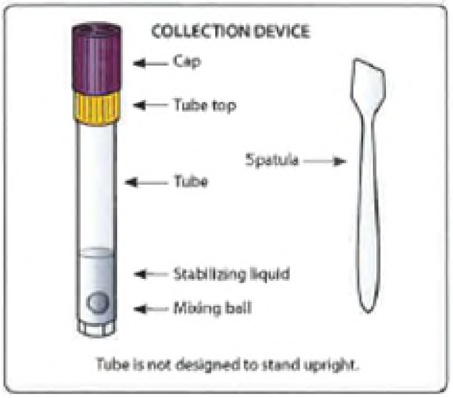

Summary and explanation of the kit:

OMNIgene•GUT provides the materials and instructions for collecting and stabilizing microbial DNA from a fecal sample.

Warnings and precautions:

- FOR EXTERNAL USE ONLY.
- Do NOT remove the yellow tube top from the tube.
- Do NOT spill the stabilizing liquid in the tube.
- Wash with water if liquid comes in contact with eyes or skin. Do NOT ingest.
- If collecting a liquid fecal sample, see separately provided user instructions.
- Small items may pose a choking hazard.

Storage: 15°C to 25°C

Ship in accordance to applicable regulations covering transport of biological specimens. See MSDS at www.dnagenotek.com

Label legend:

⌛	Collect sample by (Use by)
REF	Catalog number
🏭	Manufacturer
15°C ⁄ 25°C	Storage instructions
⚠	Caution, consult instructions for use
LOT	Lot number

USER INSTRUCTIONS

Read all instructions prior to collection

Procedure:

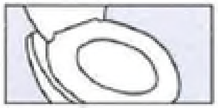

1 **IMPORTANT PREPARATIONS:**
- Empty your bladder before beginning the collection.
- Collect fecal sample free of urine or toilet water.
- Toilet paper or tissues may be required.

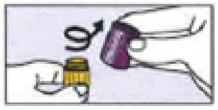

2 While holding the yellow tube top, unscrew ONLY the purple cap from the kit and set aside for later use.

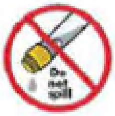

IMPORTANT:
Do NOT remove the yellow tube top.
Do NOT spill the stabilizing liquid in the tube.

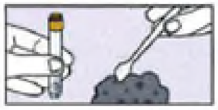

3 Use the spatula to collect a small amount of fecal sample.

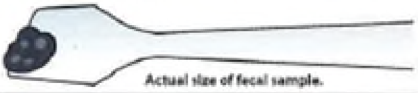

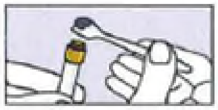

4 Transfer the fecal sample into the yellow tube top. Repeat until the sample fills the yellow tube top.

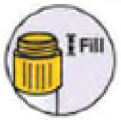

IMPORTANT: Do NOT push sample into the tube.

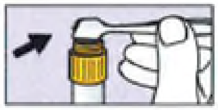

5 Scrape horizontally across the tube top to level the sample and remove any excess.

Wipe exterior of tube and top with toilet paper or tissue as needed.

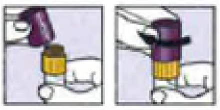

6 Pick up the purple cap with the solid end facing down and screw onto the yellow tube top until tightly closed.

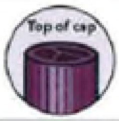

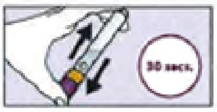

7 Shake the sealed tube as hard and fast as possible in a back and forth motion for a minimum of 30 seconds.

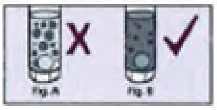

8 The fecal sample will be mixed with the stabilizing liquid in the tube; not all particles will dissolve.

IMPORTANT: Continue shaking if **large** particles remain as shown in Figure A.

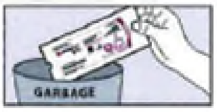

9 Place spatula in original packaging or wrap in toilet paper and discard in garbage.

IMPORTANT: Send the sample for processing following the delivery instructions supplied separately by the kit provider.

DNA GENOTEK

Superior samples
Proven performance

Made in Canada
DNA Genotek Inc.
3000 - 500 Palladium Drive
Ottawa, ON, Canada K2V 1C2

Toll-free (North America): 1.866.813.6354
Tel.: +1.613.723.5757 • Fax: +1.613.723.5057
info@dnagenotek.com
www.dnagenotek.com

Australian Sponsor: Emergo Australia, Level 20, Tower II, Darling Park, 201 Sussex Street, Sydney, NSW 2000 Australia

OMNIgene•GUT (OM-200) is not available for sale in the United States.
OMNIgene•GUT (OMR-200) is for research use only, not for use in diagnostic procedures.
*OMNIgene is a registered trademark of DNA Genotek Inc.
Some DNA Genotek products may not be available in all geographic regions, contact your sales representative for details.
All DNA Genotek protocols, white papers and application notes are available in the support section of our website at www.dnagenotek.com.

Patent (www.dnagenotek.com/legalnotices)
© 2014 DNA Genotek Inc., a subsidiary of OraSure Technologies, Inc., all rights reserved.
PD-PR-00443 Issue 5/2018-04

APPENDIX C: STUDY LOG

DIET and STOOL FREQUENCY LOG - GENERAL INSTRUCTIONS

As a part of your participation in the current study, we are requesting that you complete a study log every day.

General pointers:

- When you come to the clinic, bring your logs with you.
- Each page has room for seven days – one row should be completed for each day.
- Please make specific note of intake of any yogurt, yogurt-containing foods, or other probiotics

Example of how the top part of the log will look:

- A study team member will complete the information in this box before you leave the clinic.

COMPLETED BY STUDY TEAM	Participant Initials: JSM	Participant Research Number: 1001	Group: A

Example of how the information you enter might look:

- You or someone close to can complete the log for you, so long as the information is correct.
- List all prescription and non-prescription medications.
- The person who completes that day's entry should write his or her initials in the last column.

Day and Date	General description of food I ate:	*Did I eat yogurt or take probiotics?*	*How was my stool frequency?*	*Was a stool sample collected?*	*Medications taken*	*Initials of person filling information*
	Eggs, toast, juice<br>Ham sandwich, coke, potato chips<br>Steak, mashed potatoes, wine	○ Yes<br>☒ No	☒ Seems like baseline<br>○ 1-3 stools more than baseline<br>○ 4-6 stools more than normal<br>○ 7 or more than baseline, or incontinence	○ Yes<br>☒ No	Vitamin C,<br>Lipitor	JBC
			○ Seems like baseline			

Example of the signature line:

- When you hand over the document to the study team, they will ask to sign and date at the bottom of each log if you agree that the information is complete and correct.

At the time of handing over the document -- Participant Signature: Joseph Black Smith Date 12/18/2002

COMPLETED BY STUDY TEAM	Participant Initials:	Participant Research Number:	Group:

Day and Date	General description of food I ate:	*Did I eat yogurt or take probiotics?*	*How was my stool frequency?*	*Was a stool sample collected?*	*Medications taken*	*Initials of person filling information*
	Eggs, toast, juice<br>Ham sandwich, coke, potato chips<br>Steak, m ashed potatoes, wine	◯ Yes<br>☒ No	☒ Seems like baseline<br>◯ 1-3 stools more than baseline<br>◯ 4-6 stools more than normal<br>◯ 7 or more than baseline, or incontinence	◯ Yes<br>☒ No	Vitamin C, lipitor	JBC
		◯ Yes<br>◯ No	◯ Seems like baseline<br>◯ 1-3 stools more than baseline<br>◯ 4-6 stools more than normal<br>◯ 7 or more than baseline, or incontinence	◯ Yes<br>◯ No		
		◯ Yes<br>◯ No	◯ Seems like baseline<br>◯ 1-3 stools more than baseline<br>◯ 4-6 stools more than normal<br>◯ 7 or more than baseline, or incontinence	◯ Yes<br>◯ No		
		◯ Yes<br>◯ No	◯ Seems like baseline<br>◯ 1-3 stools more than baseline<br>◯ 4-6 stools more than normal<br>◯ 7 or more than baseline, or incontinence	◯ Yes<br>◯ No		
		◯ Yes<br>◯ No	◯ Seems like baseline<br>◯ 1-3 stools more than baseline<br>◯ 4-6 stools more than normal<br>◯ 7 or more than baseline, or incontinence	◯ Yes<br>◯ No		
		◯ Yes<br>◯ No	◯ Seems like baseline<br>◯ 1-3 stools more than baseline<br>◯ 4-6 stools more than normal<br>◯ 7 or more than baseline, or incontinence	◯ Yes<br>◯ No		

		◯ Yes<br>◯ No	◯ Seems like baseline<br>◯1-3 stools more than baseline<br>◯4-6 stools more than normal<br>◯7 or more than baseline, or incontinence	◯ Yes<br>◯ No		

STUDY DRUG MEDICATION DIARY

Please record how many capsules you take of CBM588, the time you take them and any comments here below and bring the completed Diary as well as your study drug supply, including empty bottles, to every study visit. This will help us keep track of your study drug and how well you are tolerating it.

STUDY DRUG INSTRUCTIONS:

Study Drug: CMB588
How Much: Your dose is 80 mg
How Often: You will take each dose twice daily
When: You should take your dose once in the morning and once in the evening

Cycle #: _______________
For each morning dose, take 2 packets
For each evening dose, take 2 packets

		Morning Dose		Evening Dose		
	Date	Time Taken	# of Packets Taken	Time Taken	# of Packets Taken	Comments
EXAMPLE:	2/1/2019	9:00 AM	2	8:00 PM	2	Vomited PM pill
Day 1						
Day 2						
Day 3						
Day 4						
Day 5						
Day 6						
Day 7						
Day 8						
Day 9						
Day 10						
Day 11						
Day 12						

Day 13						
Day 14						
Day 15						
Day 16						
Day 17						
Day 18						
Day 19						
Day 20						
Day 21						
Day 22						
Day 23						
Day 24						
Day 25						
Day 26						
Day 27						
Day 28						

At the time of handing over the document -- Participant Signature: ____ Date _____________

FOR STUDY TEAM USE ONLY	
Staff Initials:	
Date Dispensed:	Date Returned:
# pills/caps/tabs dispensed:	# pills/caps/tabs returned:
# pills/caps/tabs that should have been taken:	
Discrepancy Notes:	

DATA TRANSFER AGREEMENT

The Translational Genomics Research Institute (TGen) has research data hosted in an established central data repository.

Access to human genomic data will be provided to research investigators who, along with their institutions, have certified their agreement with the expectations and terms of access detailed below. It is the intent of TGen that approved users of TGen datasets recognize all restrictions on data use established by TGen.

The parties to this agreement include: the Principal Investigator (PI) requesting access to the genomic study dataset (the “Approved User”), his/her home institution as represented by the Institutional Signing Official (the “User Institution”), and TGen (the “Data Producers”). The effective date of this agreement shall be the later of a) the Project Approval Date, as specified on the Data Access Committee (DAC) approval notification, and b) the date of the last signature on this Agreement.

These terms and conditions govern access to the managed access datasets (details of which are set out in Appendix I) to which the Approved User and User Institution has requested access. The Approved User and User Institution agree to be bound by these terms and conditions.

DEFINITIONS

Approved User: The Principal Investigator for the Project.

Authorized Personnel: The individuals at the User Institution to whom TGen grants access to the Data. This includes the Approved User, the individuals listed in Appendix II and any other individuals for whom the User Institution subsequently requests access to the Data (in a subsequent, separate request approved by TGen). Details of the initial Authorized Personnel are set out in Appendix II.

Data: The managed access datasets to which the User Institution has requested access.

Data Producers: TGen and the collaborators listed in Appendix I responsible for the development, organization, and oversight of these Data.

External Collaborator: A collaborator of the Approved User, working for an institution other than the User Institution.

Project: The project for which the User Institution has requested access to these Data. A description of the Project is set out in Appendix II.

Publications: Includes, without limitation, articles published in print journals, electronic journals, reviews, books, posters and other written and verbal presentations of research.

Research Participant: An individual whose data form part of the requested Data.

Research Purposes: Shall mean research that is seeking to advance the understanding of genetics and genomics, including the treatment of disorders, and work on statistical methods that may be applied to such research.

User Institution(s): The User Institution that has requested access to the Data.

TERMS OF ACCESS

1. The User Institution agrees to only use these Data for the purpose of the Project (described in Appendix II) and only for Research Purposes. The User Institution further agrees that it will only use these Data for Research Purposes, which are within the limitations (if any) set out in Appendix I.

2. The User Institution agrees to preserve, at all times, the confidentiality of these Data. In particular, it undertakes not to use, or attempt to use these Data to compromise or otherwise breach the obligations of confidentiality of information on Research Participants. Without prejudice to the generality of the foregoing, the User Institution agrees to use at least the measures set out in Appendix I to protect these Data.

3. The User Institution agrees to protect the confidentiality of Research Participants in any research papers or publications that they prepare by taking all reasonable care to limit the possibility of identification.

4. The User Institution agrees not to link or combine these Data to other information or archived data available in a way that could re-identify the Research Participants, even if access to that data has been formally granted to the User Institution or is freely available without restriction.

5. The User Institution agrees only to transfer or disclose these Data, in whole or part, or any material derived from these Data, to the Authorized Personnel. Should the User Institution wish to share these Data with an External Collaborator, the External Collaborator must complete a separate application for access to these Data.

6. The User Institution agrees that the Data Producers, and all other parties involved in the creation, funding or protection of these Data: a) make no warranty or representation, express or implied as to the accuracy, quality or comprehensiveness of these Data; b) exclude to the fullest extent permitted by law all liability for actions, claims, proceedings, demands, losses (including but not limited to loss of profit), costs, awards damages and payments made by the User Institution that may arise (whether directly or indirectly) in any way whatsoever from the User Institution's use of these Data or from the unavailability of, or break in access to, these Data for whatever reason and; c) bear no responsibility for the further analysis or interpretation of these Data.

7. The User Institution agrees to follow the *Fort Lauderdale Guidelines* and the *Toronto Statement*. This includes but is not limited to recognizing the contribution of the Data Producers and including a proper acknowledgement in all reports or publications resulting from the use of these Data.

8. The User Institution agrees to follow the Publication Policy in Appendix III. This includes respecting the moratorium period for the Data Producers to publish the first peer-reviewed report describing and analyzing these Data.

9. The User Institution agrees not to make intellectual property claims on these Data and not to use intellectual property protection in ways that would prevent or block access to, or use of, any element of these Data, or conclusion drawn directly from these Data.

10. The User Institution can elect to perform further research that would add intellectual and resource capital to these data and decide to obtain intellectual property rights on these downstream discoveries. In this case, the User Institution agrees to implement licensing policies that will not obstruct further research and to follow the U.S. National Institutes of Health *Best Practices for the Licensing of Genomic Inventions* *(2005)* in conformity with the Organization for Economic Co-operation and Development *Guidelines for the Licensing of the Genetic Inventions* *(2006)*.

11. The User Institution agrees to destroy/discard the Data held, once it is no longer used for the Project, unless obliged to retain the Data for archival purposes in conformity with audit or legal requirements.

12. The User Institution will notify TGen in writing within 30 days of any changes or departures of Authorized Personnel.

13. The User Institution will notify TGen in writing prior to any significant changes to the protocol for the Project.

14. The User Institution will notify TGen in writing as soon as it becomes aware of a breach of the terms or conditions of this agreement.

15. TGen or the User Institution may terminate this agreement at any time, and for any reason, by written notice to the other party. If this agreement is terminated, the User Institution shall destroy any Data held, including copies and backup copies. This clause does not prevent the User Institution from retaining these Data for archival purpose in conformity with audit or legal requirements.

16. The User Institution accepts that it may be necessary for the Data Producers to modify the terms of this agreement from time to time. As an example, such modifications may include specific provisions relating to the Data required by Data Producers other than TGen. In the event that changes are required, the Data Producers or their appointed agent will contact the User Institution to inform it of the changes, and the User Institution may elect to accept the changes or terminate the agreement.

17. If requested, the User Institution will allow data security and management documentation to be inspected by TGen to verify that it is complying with the terms of this agreement.

18. The User Institution agrees to distribute a copy of these terms to all Authorized Personnel and require the Authorized Personnel to comply with the terms of this agreement.

19. This agreement (and any dispute, controversy, proceedings or claim of whatever nature arising out of this agreement or its formation) shall be construed, interpreted and governed by the laws of the state of New York, without reference to its conflicts of law provisions.

Agreed for User Institution (Signatory Official)

**Signature:**	
**Name:**	
**Title:**	
**Date:**	

Principal Investigator

I confirm that I have read and understood this Agreement.

**Signature:**	
**Name:**	
**Title:**	
**Date:**	

Agreed for TGen

**Signature:**	
**Name:**	**Stephanie Buchholtz, PhD**
**Title:**	**Sr. Director, Office of Research Compliance & Quality Mgmt**
**Date:**	

APPENDIX I – DATASET DETAILS
APPENDIX II —PROJECT DETAILS
APPENDIX III — PUBLICATION POLICY

APPENDIX I – DATASET DETAILS

Dataset reference (TGen Study ID and Dataset Details)

[insert name of data set] Dataset maintained by the Translational Genomics Research Institute.

Name of project that created the dataset

[insert study title]

Names of other data producers/collaborators

[insert collaborators]

Specific limitations on areas of research

[insert limitations such as consent limitations or "No specific limitations set"]

Minimum protection measures required

The Approved User, their User Institution and all Authorized Users agree to handle the data according to the current *NIH Security Best Practices for Controlled-Access Data Subject to the NIH Genomic Data Sharing (GDS) Policy* *(09MAR2015)*.

File access

Data can be held in unencrypted files on an institutional computer system, with Unix user group read/write access for one or more appropriate groups but not Unix world read/write access behind a secure firewall. Laptops holding these data should have password protected logins and screen locks (set to lock after 5 min of inactivity). If held on USB keys or other portable hard drives, the data must be encrypted.

APPENDIX II – PROJECT DETAILS (to be completed by the Approved User)

Details of dataset requested i.e., EGA Study and Dataset Accession Number

[insert Data set name or ID #] Dataset maintained by the Translational Genomics Research Institute.

Name of Principal Investigator	***Email***	***Job Title***	***User Institution***

Brief abstract of the Project in which the Data will be used (500 words max)

All Individuals who the User Institution requests to be named as Authorized Personnel (add rows as needed)

Name of Authorized Personnel	***Email***	***Job Title***	***Supervisor****

All Individuals that should have an account created at the EGA

**Name of Authorized User**	**Email**	**Job Title**

APPENDIX III – PUBLICATION POLICY

In any publications based on these Data, please describe how the Data can be accessed, including the name of the data set and its accession numbers, and acknowledge its use in a form agreed by the User Institution with TGen.
