# Supplementary material for: Nivolumab plus ipilimumab with or without live bacterial supplementation in metastatic renal cell carcinoma: a randomized phase 1 trial
Source: Nat Med. 2022 Feb 28;28(4):704–12. doi: 10.1038/s41591-022-01694-6 (PMC9018425; doi:10.1038/s41591-022-01694-6)
Supplement: Supplementary file 2 — Reporting Summary [file 41591_2022_1694_MOESM2_ESM.pdf]

nature portfolio

Corresponding author(s): Sumanta Pal, MD.

Last updated by author(s): 2021-15-12

Reporting Summary

Nature Portfolio wishes to improve the reproducibility of the work that we publish. This form provides structure for consistency and transparency in reporting. For further information on Nature Portfolio policies, see our Editorial Policies and the Editorial Policy Checklist.

Statistics

For all statistical analyses, confirm that the following items are present in the figure legend, table legend, main text, or Methods section.

n/a	Confirmed	
☐	☒	The exact sample size (*n*) for each experimental group/condition, given as a discrete number and unit of measurement
☐	☒	A statement on whether measurements were taken from distinct samples or whether the same sample was measured repeatedly
☐	☒	The statistical test(s) used AND whether they are one- or two-sided<br>*Only common tests should be described solely by name; describe more complex techniques in the Methods section.*
☐	☒	A description of all covariates tested
☒	☐	A description of any assumptions or corrections, such as tests of normality and adjustment for multiple comparisons
☐	☒	A full description of the statistical parameters including central tendency (e.g. means) or other basic estimates (e.g. regression coefficient) AND variation (e.g. standard deviation) or associated estimates of uncertainty (e.g. confidence intervals)
☐	☒	For null hypothesis testing, the test statistic (e.g. *F*, *t*, *r*) with confidence intervals, effect sizes, degrees of freedom and *P* value noted<br>*Give P values as exact values whenever suitable.*
☒	☐	For Bayesian analysis, information on the choice of priors and Markov chain Monte Carlo settings
☒	☐	For hierarchical and complex designs, identification of the appropriate level for tests and full reporting of outcomes
☒	☐	Estimates of effect sizes (e.g. Cohen's *d*, Pearson's *r*), indicating how they were calculated

Our web collection on statistics for biologists contains articles on many of the points above.

Software and code

Policy information about availability of computer code

Data collection	Clinical data was collected in a protected institutional database.
Data analysis	R software 4.1.1, SPSS 21.0, GraphPad Prism 8.4.3, MetaPhlAn 3.0 , HuMAnN 3.0

For manuscripts utilizing custom algorithms or software that are central to the research but not yet described in published literature, software must be made available to editors and reviewers. We strongly encourage code deposition in a community repository (e.g. GitHub). See the Nature Portfolio guidelines for submitting code & software for further information.

Data

Policy information about availability of data

All manuscripts must include a data availability statement. This statement should provide the following information, where applicable:

- Accession codes, unique identifiers, or web links for publicly available datasets
- A description of any restrictions on data availability
- For clinical datasets or third party data, please ensure that the statement adheres to our policy

Metagenomic data from stool sufficient to replicate the analyses presented herein will be deposited in Translational Genomics Research Institute (TGen) and will be available upon request. Authors defer depositing the participant genomic data in national and international public repositories due to institutional policies, and the absence of statements in patient consent forms which would have allowed controlled access distribution and genomic data availability. De-identified individual participant whole metagenome libraries and clinical data that underlie the results reported in this article are available for transfer on a specific secure server housed at TGen. Interested investigators can obtain and certify the data transfer agreement (DTA) and submit requests to the principal investigator, Sumanta K. Pal, MD (spal@coh.org). Proposals will be vetted by the TGen Data Access Committee. Investigators/institutions who consent to the terms of the DTA form, including but not limited to the use of these data for the purpose of a specific project and only for research purposes, protect the confidentiality of the data and limit the

possibility of identification of participants in any way whatsoever for the duration of the agreement will be granted access. TGen will then facilitate the transfer of the requested de-identified data. This mechanism is expected to be via an Aspera High Speed File Transfer Server at the time of this publication, but TGen reserves the right to change the specific transfer method at any time, provided appropriate levels of access authorization and control can be maintained.

Field-specific reporting

Please select the one below that is the best fit for your research. If you are not sure, read the appropriate sections before making your selection.

☒ Life sciences ☐ Behavioural & social sciences ☐ Ecological, evolutionary & environmental sciences

For a reference copy of the document with all sections, see nature.com/documents/nr-reporting-summary-flat.pdf

Life sciences study design

All studies must disclose on these points even when the disclosure is negative.

Sample size	With a cumulative sample size of 30 patients (randomized in a 2:1 fashion), we would have 80% power to detect a one standard deviation change in specific Bifidobacterium spp. between study arms using a Mann-Whitney U-test with a one-sided type I error of 0.05.
Data exclusions	All data corresponding to one patient originally randomized into the nivolumab/ipilimumab plus CBM588 arm was excluded. The patient was deemed ineligible after treatment initiation because tissue-based next-generation sequencing performed as part of routine clinical care showed genomic alterations pathognomonic for sarcoma
Replication	N/A
Randomization	Patients were randomized in a 2:1 fashion using a permuted block design to receive either nivolumab/ipilimumab with CBM588 or nivolumab/ipilimumab alone
Blinding	Investigators were not blinded to group allocation during data collection as the protocol demanded the use of dietary and medication logs and was not placebo controlled. However, analysis of the samples collected was performed in a blinded fashion.

Reporting for specific materials, systems and methods

We require information from authors about some types of materials, experimental systems and methods used in many studies. Here, indicate whether each material, system or method listed is relevant to your study. If you are not sure if a list item applies to your research, read the appropriate section before selecting a response.

Materials & experimental systems

n/a	Involved in the study	
☐	☒	Antibodies
☒	☐	Eukaryotic cell lines
☒	☐	Palaeontology and archaeology
☒	☐	Animals and other organisms
☐	☒	Human research participants
☐	☒	Clinical data
☒	☐	Dual use research of concern

Methods

n/a	Involved in the study	
☒	☐	ChIP-seq
☒	☐	Flow cytometry
☒	☐	MRI-based neuroimaging

Antibodies

Antibodies used	Fc III/IIR-specific antibody was used to block nonspecific binding.
Validation	Commercially available Fc III/IIR-specific antibodies that have been validated by BD and Biolegend were used in the study.

Human research participants

Policy information about studies involving human research participants

Population characteristics	Patients included in this study had histologically confirmed clear cell renal cell carcinoma and/or sarcomatoid histology. Patients had to be age 18 or older and have histologically confirmed mRCC with no prior systemic therapy (prior adjuvant therapy was allowed unless with a immune checkpoint inhibitor). Patients were required to have intermediate- or poor-risk disease based on International mRCC Database Consortium (IMDC) criteria. Measurable disease by Response Evaluation Criteria in Solid Tumors (RECIST) version 1.1 was required. A total of 30 patients were randomized and started protocol-based treatment between April 22, 2019 and December 30, 2020 (see Extended Data Figure 1 for CONSORT diagram). One patient originally randomized into the nivolumab/ipilimumab plus CBM588 arm was deemed ineligible after treatment initiation because tissue-based next-generation sequencing performed as part of routine clinical care showed genomic alterations

pathognomonic for sarcoma. Ultimately, 29 patients were included in the final analysis. Baseline patient characteristics are shown in Table 1. The median age of the overall cohort was 66 (range, 45-90) and the majority of the patients (72%) were male. Patients with sarcomatoid histology comprised 34% of the study cohort. The most common metastatic sites were lung, lymph nodes and bone.

Recruitment

Participants were approached during routine clinical visits and screened for eligibility/consented if inclusion criteria were met and no exclusion criteria were identified. We do not anticipate a bias in recruitment of participants in this study.

Ethics oversight

The study (NCT03829111) was approved by the US Food and Drug Administration and by the City of Hope Institutional Review Board. Patients were required to supply written informed consent prior to participating. All study procedures were undertaken in accordance with the Declaration of Helsinki.

Note that full information on the approval of the study protocol must also be provided in the manuscript.

Clinical data

Policy information about clinical studies

All manuscripts should comply with the ICMJE guidelines for publication of clinical research and a completed CONSORT checklist must be included with all submissions.

Clinical trial registration

The study (NCT03829111) was approved by the US Food and Drug Administration

Study protocol

The full trial protocol will be made available as part of the supplemental material

Data collection

All data was collected at the City of Hope Comprehensive Cancer Center in Duarte California.
Recruitment: 30 patients were recruited randomized from April of 2019 to November of 2020.
Data Collection: Collection from patient related data was performed from April 22,2019 to April 15,2021.

Outcomes

Primary Endpoint
Change in Bifidobacterium composition of stool from baseline to week 12 of therapy on the CBM588+nivolumab/ipilimumab vs nivolumab/ipilimumab alone.

Secondary Endpoints
Comparison of the Shannon index (a measure of microbial diversity) from baseline to week 12 of therapy on the CBM588 +nivolumab/ipilimumab vs nivolumab/ipilimumab alone.
Best overall response, by RECIST criteria, with nivolumab/ipilimumab alone vs nivolumab/ipilimumab with CBM588
Progression-free survival (PFS), assessed as the duration of time from enrollment to progression, with nivolumab/ipilimumab alone vs nivolumab/ipilimumab with CBM588
Comparison of the proportion of circulating Tregs at baseline to levels of circulating Tregs with nivolumab/ipilimumab alone vs nivolumab/ipilimumab with CBM588
Comparison of the proportion of circulating MDSCs with nivolumab/ipilimumab alone versus nivolumab/ipilimumab with CBM588
Comparison of IL-6, IL-8 and other cytokines/chemokines with nivolumab/ipilimumab alone versus nivolumab/ipilimumab with CBM588
Comparison of toxicities such as diarrhea and nausea using CTCAE v5 criteria with nivolumab/ipilimumab alone versus nivolumab/ipilimumab with CBM588

Assessment
Patients were required to have computerized tomography of the chest, abdomen and pelvis at baseline; technetium bone scan and central nervous system imaging was performed as clinically indicated. Patients were assessed with imaging at 12-week intervals thereafter, with follow-up until termination of protocol-based therapy or death. Safety evaluations were conducted on three-week intervals for 12 weeks, followed by monthly evaluation. Radiographic response was assessed using RECIST version 1.1.

Stool was collected from patients at baseline and 12 weeks. Patients were provided with a stool collection kit (OMNIgene Gut; DNA Genotek, Ottawa, CN); samples were mailed to TGen North within hours of collection. DNA was extracted from stool samples using the MagMax PowerMicrobiome extraction kit using the KingFisher Flex magnetic purification system (ThermoFisher, Waltham, MA) with prior bead beating using a TissueLyser (Qiagen, Valencia, CA). Bacterial load was quantitated using the BactQuant TaqMan assay.18 Whole metagenome libraries were constructed using a KAPA HyperPrep Library Kit (Roche, Indianapolis, IN), and normalized, pooled and sequenced on the Illumina NextSeq platform (2 x 150 bp). Reads were trimmed using Trimmomatic to remove adapters and low quality bases and reads.19 Samples that passed quality were taxonomically profiled using MetaPhlAn3 and output was merged to retain species level assignments
